# Short‐ and Long‐Term Control of Hypertension After Bariatric Surgery: A 10‐Year Prospective Study

**DOI:** 10.1002/mco2.71097

**Published:** 2026-10-29

**Authors:** Xiaoxiao Zhong, Yaoquan Cao, Xulong Sun, Pengzhou Li, Weizheng Li, Hong Yuan, Shaihong Zhu, Liangping Wu, Hua Meng, Yao Lu, Liyong Zhu

**Affiliations:** ^1^ Department of Bariatric and Metabolic Surgery The Third Xiangya Hospital of Central South University Changsha Hunan China; ^2^ Clinical Research Center The Third Xiangya Hospital of Central South University Changsha Hunan China; ^3^ Department of Cardiology The Third Xiangya Hospital of Central South University Changsha Hunan China; ^4^ Furong Laboratory Central South University Changsha Hunan China; ^5^ Department of Metabolic Surgery The Jinshazhou Hospital of Guangzhou University of Chinese Medicine Guangzhou China; ^6^ Department of General Surgery The China‐Japan Friendship Hospital Beijing China

**Keywords:** bariatric surgery, blood pressure, hypertension, obesity

## Abstract

Current guidelines support bariatric surgery (BS) as an effective treatment mainly for patients with severe obesity and poorly controlled hypertension. However, evidence in non‐severely obese Asian populations remains limited. This multicenter prospective cohort study included 248 Chinese patients with obesity and hypertension who underwent BS between 2010 and 2019 and were followed for up to 10 years. An exploratory mechanistic substudy (*n* = 14) evaluated 24‐h ambulatory blood pressure (BP), various biomarkers, and urinary electrolytes before and after BS. Compared with baseline, adjusted mean systolic blood pressure (SBP) was lower by 16.4, 14.8, 15.1, 18.0, and 15.2 mmHg, while diastolic blood pressure (DBP) decreased by 11.4, 9.1, 9.9, 10.5, and 11.4 mmHg at 3 months, 6 months, and 1, 5, 10 years postoperatively. During postoperative Days 1 to 7 (POD1‐7), mean SBP and DBP dropped by 8.6 and 10.4 mmHg, and the proportion achieving BP < 140/90 mmHg increased to 62.9% and remained at 70.8% even 10 years later. The substudy has observed that early BP reduction was accompanied by increased electrolytes and water excretion, as well as a transient suppression of renin‐angiotensin‐aldosterone system (RAAS)‐related biomarkers, providing a direction for future in‐depth exploration. These findings suggest that BS in Chinese patients was followed by rapid BP reductions, with lower BP observed among the smaller subsets assessed longer term, including those without severe obesity.

**Trial Registration**: NCT07183137.

## Graphical abstract

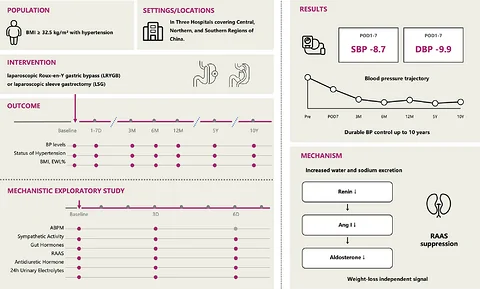

## 1 Introduction

Bariatric surgery (BS) has been recognized for more than three decades as an effective treatment for obesity and its related comorbidities, including hypertension. Several randomized clinical trials have demonstrated that BS benefits hypertension, leading to a reduction in both blood pressure (BP) and the need for antihypertensive medications reduction [1, 2, 3]. Consistently, international and Chinese guidelines recommend BS for controlling BP in patients with severe obesity [the 2024 Chinese obesity guideline defining severe obesity as body mass index (BMI) ≥ 37.5 kg/m^2^] combined with hypertension [4, 5, 6]. Notably, preliminary work to expand the clinical indications for BS in China remains incomplete, as the postoperative BP reduction trend in the Chinese population remains unclear.

Furthermore, although clinical attention often focuses on individuals with severe obesity, it is not uncommon for those with mild to moderate obesity (BMI < 37.5 kg/m^2^) to experience obesity‐related cardiovascular events (CVD) [7]. This population is particularly prevalent in Asian populations, including China, characterized by disproportionately high rates of visceral fat accumulation and central obesity despite relatively low BMI levels [8, 9]. Over half of Chinese adults with hypertension are overweight or mildly obese [10, 11, 12], underscoring the urgent need to address the cardiovascular burden associated with the low‐BMI obesity phenotype. Although the GATEWAY trial (Gastric Bypass to Treat Obese Patients With Steady Hypertension [1], the only randomized trial to compare the effects on BP reduction between BS vs. medical therapy) included a part of Brazilian subjects with mild obesity, most published studies have focused on hypertension resolution in patients with severe obesity. Concurrently, many prior studies included small sample sizes [13, 14], adopted retrospective designs [15, 16], or had insufficient follow‐up durations to fully characterize long‐term BP trend. In other words, evidence describing the complete process of BP changes from immediate postoperative to long‐term follow‐up remains scarce.

In this study, we aimed to determine the effect of BS on the entire course of BP change, including short term, intermediate term, and long term, in a multicenter cohort based on a Chinese population, while also observing the significant BP‐lowering benefits of BS in Chinese patients with mild obesity. In addition, we conducted a mechanistic exploratory study involving 24‐h ambulatory BP monitoring and early postoperative physiological assessments to investigate potential weight‐independent pathways contributing to the rapid postoperative decline in BP, which is expected to provide a theoretical basis for future in‐depth studies on the weight‐independent mechanisms of BS for the treatment of hypertension.

## 2 Results

### 2.1 Participant Characteristics

A total of 1788 patients were enrolled between September 2010 and December 2019 (Figure S1). Among them, 248 patients were included in the final analysis. The overall experimental design is shown in Figure 1. The number of patients who completed follow‐up assessments at each time point was clearly recorded and presented in Figure 2 and Tables S1 and S2. Baseline data are summarized in Table 1. The average age was 36.83 ± 10.86 years. Females made up 49.60% of the cohort. The mean BMI was 38.02 ± 7.35 kg/m^2^. The average preoperative systolic blood pressure (SBP) and diastolic blood pressure (DBP) were 142.70 ± 13.96 mmHg and 93.93 ± 10.94 mmHg, respectively.

**FIGURE 1:**
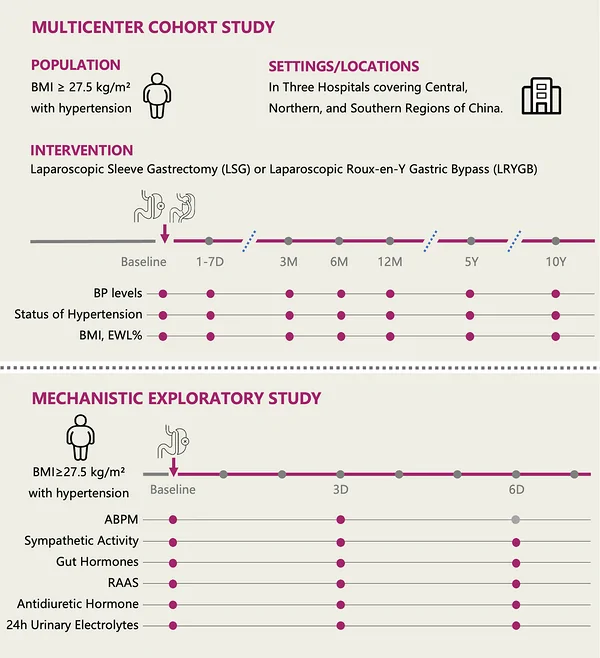
Study flow diagram.

**FIGURE 2:**
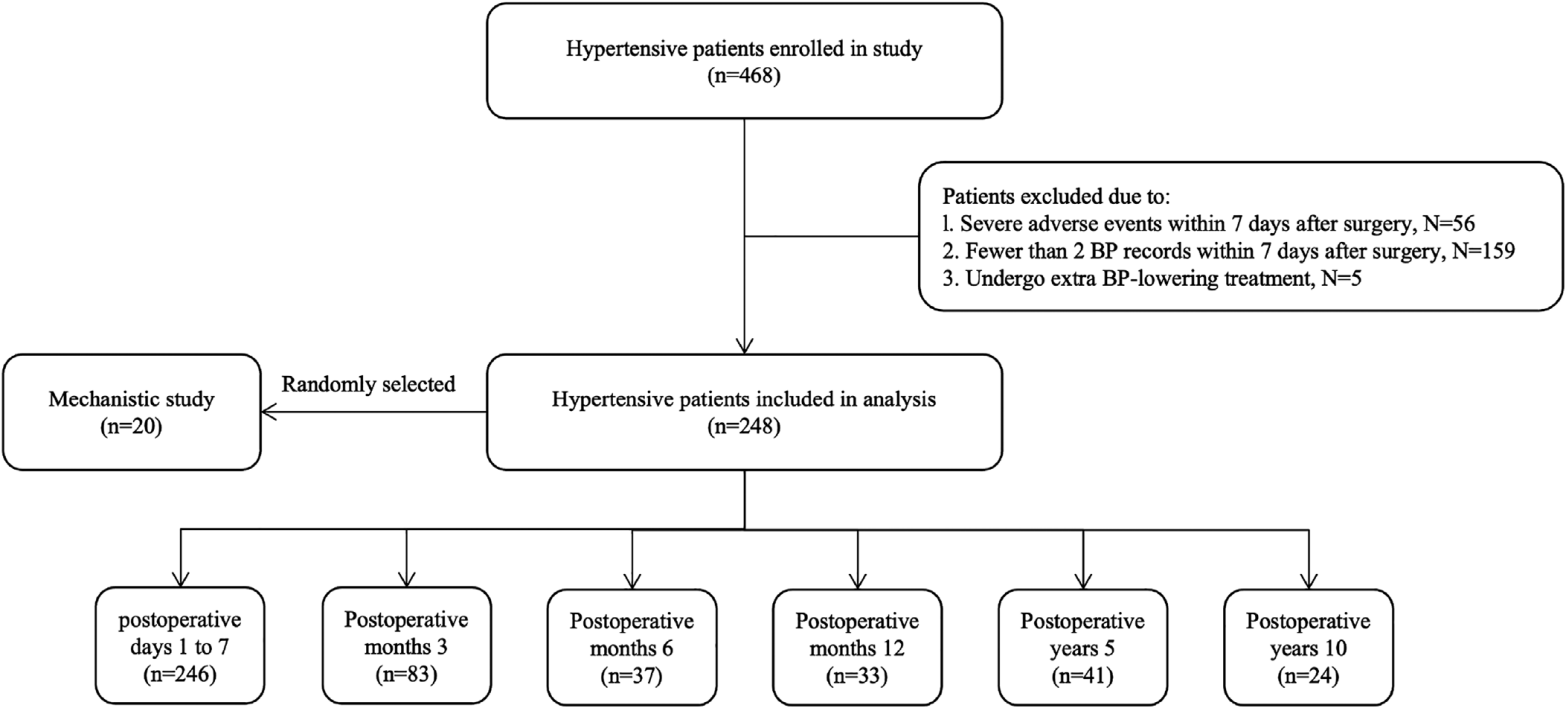
Flow chart of enrolled patients in the study.

**TABLE 1:** Baseline characteristics of hypertensive patients.

Characteristic	Total (*n* = 248)
Age, years	36.83 ± 10.86
Sex, *n* (%)	
Male	125 (50.4)
Female	123 (49.6)
BMI, kg/m^2^	38.02 ± 7.35
WC, cm	118.12 ± 15.98
SBP, mmHg	142.70 ± 13.96
DBP, mmHg	93.93 ± 10.94
Hb, g/L	145.10 ± 17.00
RBC count, 10^12^/L	5.00 ± 0.70
Albumin, g/L	44.41 ± 4.16
Scr, µmol/L	66.31 ± 20.10
UA, µmol/L	410.76 ± 111.11
TC, mmol/L	4.91 ± 1.01
TG, mmol/L	2.65 ± 2.20
HDL‐C, mmol/L	1.07 ± 0.28
LDL‐C, mmol/L	2.82 ± 0.86
FBG, mmol/L	7.59 ± 3.42
Fasting insulin, µU/mL	28.73 ± 24.41
HbA1c, %	7.35 ± 1.90
Use of BP‐lowering drugs, *n* (%)	
No	164 (66.1)
Yes	84 (33.9)
Surgical procedure, *n* (%)	
LRYGB	99 (39.9)
LSG	149 (60.1)
Study center, *n* (%)	
Third Xiangya Hospital	125 (50.4)
Guangzhou University of Chinese Medicine Jinshazhou Hospital	99 (39.9)
China–Japan Friendship Hospital	24 (9.7)

*Note*: Characteristic values are presented as mean ± standard deviation (SD) or *n* (%).

Abbreviations: DBP, diastolic blood pressure; FBG, fasting blood glucose; Hb, hemoglobin; HDL‐C, high‐density lipoprotein cholesterol; LDL‐C, low‐density lipoprotein cholesterol; LRYGB, laparoscopic Roux‐en‐Y gastric bypass; LSG, laparoscopic sleeve gastrectomy; BMI, body mass index; RBC, red blood cell; SBP, systolic blood pressure; Scr, serum creatinine; TC, total cholesterol; TG, triglyceride; UA, uric acid; WC, waist circumference.

### 2.2 Intermediate‐Term and Long‐Term Effect of Bariatric Surgery on Blood Pressure

The mean SBP at postoperative 3 months, 6 months, and 1 year were 126.66 ± 14.57, 124.54 ± 16.59, and 126.15 ± 17.74 mmHg, and the mean DBP were 83.37 ± 10.25, 82.28 ± 12.14, and 82.38 ± 10.06 mmHg. The effect was maintained until postoperative 5–10 years, with the SBP of 123.76 ± 15.83 and 124.83 ± 13.08 mmHg in postoperative 5 years and DBP of 87.17 ± 8.05 and 81.58 ± 9.93 mmHg in postoperative 10 years (Table S2). Postoperative BP trajectories show significant improvement in BP over the medium to long term (Figure 3A,B). The mean BP at postoperative 1, 5, and 10 years was not significantly altered compared to the BP at postoperative 6 months (Figure 3A,B and Table S2). After adjustment for potential confounding factors in the preoperative, the long‐term maintenance of BP decline postoperatively were consistent from linear mixed‐effects models (LMMs) results: compared with preoperative, SBP remained significantly reduced at 3 and 6 months postoperatively (−16.35 mmHg and −14.84 mmHg, respectively; all *p* < 0.001), with this effect persisting at 1, 5, and 10 years postoperatively (−15.06 mmHg, −17.98 mmHg, and −15.17 mmHg, respectively; all *p* < 0.001), and DBP showed a similar trend (Table 2). Compared to the SBP change during postoperative Days 1 to 7 (POD1–7), significantly greater reductions in SBP were observed at 3 months, 5 years, and 10 years postoperatively. In contrast, changes in DBP at each follow‐up time point were not significantly different from the DBP change during POD1‐7 (Table 2). Compared with baseline (14.92%), the proportion of patients whose BP was controlled at <140/90 mmHg increased significantly during the 10‐year follow‐up period, reaching 62.90% at 1–7 days, 73.49% at 3 months, 61.11% at 6 months, 72.73% at 1 year, 68.29% at 5 years, and 70.83% at 10 years after surgery (Table S2).

**FIGURE 3:**
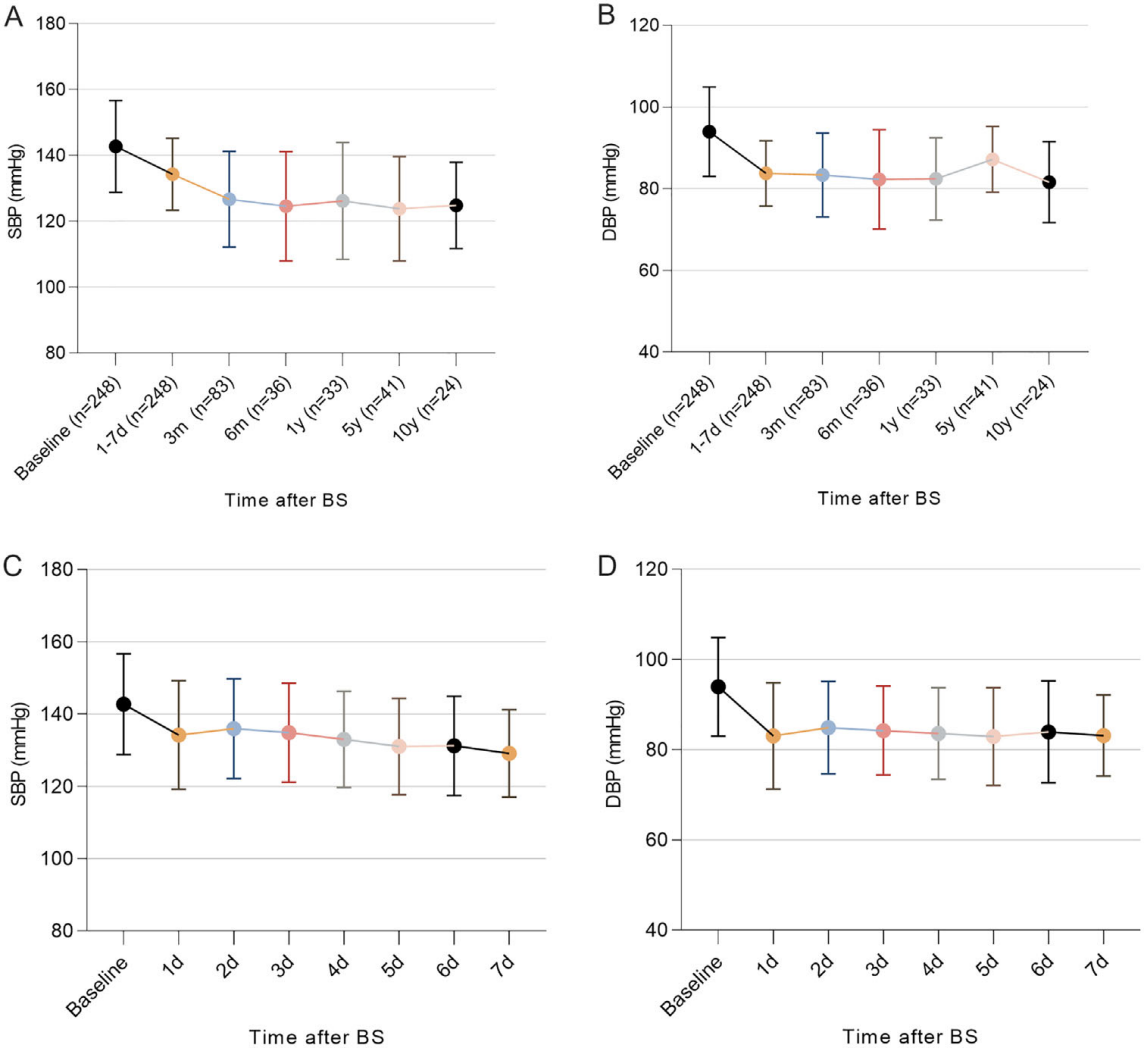
The trajectory of blood pressure in the long and short term after bariatric surgery. (A) Trajectory of SBP from POD1‐7 to postoperative 12 months; (B) trajectory of DBP from POD1‐7 to postoperative 12 months; (C) trajectory of SBP from postoperative Days 1–7; (D) trajectory of DBP from postoperative days 1–7. DBP, diastolic blood pressure; SBP, systolic blood pressure; POD1‐7, postoperative days 1 to 7.

**TABLE 2:** Long‐term effects of bariatric surgery on blood pressure.

Follow‐up time	Adjusted SBP change, mmHg (95% CI)	*p* value	Adjusted DBP change, mmHg (95% CI)	*p* value
1‐7 Days	−8.62 (−10.43, −6.81)	< 0.001	−10.43 (−11.75, −9.12)	< 0.001
3 Months	−16.35 (−19.03, −13.68)^####^	< 0.001	−11.41 (−13.35, −9.47)	< 0.001
6 Months	−14.84 (−18.54, −11.14)^##^	< 0.001	−9.08 (−11.80, −6.37)	< 0.001
1 Year	−15.06 (−19.00, −11.12)^##^	< 0.001	−9.85 (−12.67, −7.03)	< 0.001
5 Years	−17.98 (−21.57, −14.39)^####^	< 0.001	−10.48 (−13.08, −7.87)	< 0.001
10 Years	−15.17 (−19.85, −10.49)^###^	< 0.001	−11.35 (−14.75, −7.96)	< 0.001

*Note*: Changes over long term in blood pressure post bariatric surgery were estimated using maximum‐likelihood linear mixed‐effects models with participant‐level random intercepts. Models were adjusted for age, sex, baseline BMI, baseline antihypertensive medication use, the corresponding baseline BP, baseline total cholesterol, study center, surgical procedure, and calendar year of surgery. *p* value, compared with baseline.

Abbreviations: 95% CI, change from baseline 95% confidence interval; DBP, diastolic blood pressure; SBP, systolic blood pressure.

^##^ ***p*** < 0.0; ^###^ *p* < 0.001; ^####^ *p* < 0.0001, compared with 1–7 days.

### 2.3 Short‐Term Effect of Bariatric Surgery on Blood Pressure

Compared to preoperative BP, BP was significantly lower in 1–7 days postoperatively, with SBP of 134.2 ± 15.02, 135.99 ± 13.79, 134.87 ± 13.71, 133 ± 13.26, 131.04 ± 13.33, 131.19 ± 13.77, and 129.15 ± 12.1 mmHg and DBP of 83.04 ± 11.78, 84.88 ± 10.22, 84.23 ± 9.88, 83.58 ± 10.18, 82.91 ± 10.82, 83.96 ± 11.28, and 83.15 ± 9 mmHg, respectively (Table S3). BP has already begun to decline in the short‐term following surgery (Figure 3C,D and Table S3). After adjusting for potential preoperative confounding factors, we still observed an immediate and significant decrease in both systolic and DBP immediately after BS: compared with preoperative, a significant decrease in SBP was observed at POD1 (−8.6 mmHg, 95% confidence interval [CI]: −10.6 to −6.6; *p* < 0.001), and this trend persisted from POD2 to POD7 (−7.0 to −14.4 mmHg; all *p* values < 0.001); DBP also showed a similar trend (Table 3). Then, compared to the SBP change on postoperative Day 1, a significantly greater reduction was observed on Day 7. No significant differences were found at other time points. For DBP, changes from postoperative Days 2 to 7 were not significantly different from the change on Day 1 (Table 3).

**TABLE 3:** Short‐term effects of bariatric surgery on blood pressure.

Follow‐up time	Adjusted SBP change, mmHg (95% CI)	*p* value	Adjusted DBP change, mmHg (95% CI)	*p* value
POD_1_	−8.60 (−10.57, −6.63)	< 0.001	−11.03 (−12.56, −9.49)	< 0.001
POD_2_	−7.00 (−9.05, −4.95)	< 0.001	−8.66 (−10.26, −7.07)	< 0.001
POD_3_	−8.69 (−10.84, −6.54)	< 0.001	−9.73 (−11.40, −8.06)	< 0.001
POD_4_	−10.15 (−12.34, −7.96)	< 0.001	−10.47 (−12.17, −8.76)	< 0.001
POD_5_	−12.48 (−14.87, −10.09)	< 0.001	−12.09 (−13.95, −10.23)	< 0.001
POD_6_	−12.32 (−14.99, −9.66)	< 0.001	−10.61 (−12.68, −8.54)	< 0.001
POD_7_	−14.39 (−17.51, −11.27)	< 0.001	−11.67 (−14.09, −9.25)	< 0.001

*Note*: Change over short time in blood pressure post bariatric surgery were estimated using maximum‐likelihood linear mixed‐effects models with participant‐level random intercepts. Models were adjusted for age, sex, baseline BMI, baseline antihypertensive medication use, the corresponding baseline BP, baseline total cholesterol, study center, surgical procedure, and calendar year of surgery.

Abbreviations: 95%CI, change from baseline 95% confidence interval; DBP, diastolic blood pressure; SBP, systolic blood pressure.

### 2.4 Effect of Bariatric Surgery on Blood Pressure in Different BMI and Procedures Groups

The BP control effect of BS on different BMI groups did not differ significantly in both the long‐term and short‐term (SBP: interaction between time and BMI groups, *p* = 0.736; DBP: interaction between time and BMI groups, *p* = 0.912), as shown in Table S4 for the specific levels of BP reduction. In addition, the longitudinal change of SBP differed significantly between surgical procedures (interaction between time and surgical procedure, *p* = 0.012; Table S5). Compared with laparoscopic sleeve gastrectomy (LSG), laparoscopic Roux‐en‐Y gastric bypass (LRYGB) was associated with a greater early reduction in SBP, particularly on POD1 and POD5.

### 2.5 Blood Pressure Changes After Bariatric Surgery by Baseline Antihypertensive Medication Use

We further stratified participants based on their baseline use of antihypertensive medications to investigate between‐group differences in the effect of BS on BP reduction. Compared with the group receiving medication at baseline, the group not receiving medication exhibited a greater and more sustained reduction in BP (Table S6). A significant interaction indicated that baseline antihypertensive medication use had a substantial impact on the overall postoperative longitudinal blood pressure trajectory (SBP: interaction between time and antihypertensive medication use, *p* = 0.006; DBP: interaction between time and antihypertensive medication use, *p* < 0.001). These results suggest that BS was associated with not only a potent and sustained reduction in BP in the absence of medication but also a further BP‐lowering effects of medication when used in combination with surgery.

### 2.6 The Effects of Bariatric Surgery on Blood Pressure in Patients With Different Degrees of Weight Loss

Given that most previous studies have confirmed that body weight does not fluctuate significantly during the first week after surgery, particularly during Days 1–3 [17, 18]. In our analysis of the short‐term effects of BS on BP, we found that these effects began to appear on the first day after surgery, indicating that the effect is virtually unrelated to weight loss (Table 3).

In the time‐varying covariate analysis incorporating percentage total weight loss (TWL%) conducted between 3 months and 10 years post‐surgery, neither the overall time‐by‐TWL% interaction nor the average marginal association between time‐varying TWL% and blood pressure change was statistically significant (Table S7). Subsequently, since weight loss gradually stabilizes 1 year after BS [19, 20], we conducted a subgroup analysis stratified by the median TWL% at 1‐year post‐surgery (26.09%) to assess the effect of the BS on BP between the two groups. SBP decreased significantly from baseline levels throughout the follow‐up period in both the lower and higher TWL% groups. Here, no trend was observed suggesting that higher TWL% groups experienced greater long‐term reductions in BP (SBP: interaction between time and TWL%, *p* = 0.311; Table S8). These findings suggest that suggesting that the sustained postoperative reductions in BP were not clearly dependent on the magnitude of concurrent weight loss and may partly involve mechanisms beyond weight loss alone.

### 2.7 Sensitivity Analysis of the Effects of Bariatric Surgery on Blood Pressure

Because the number of cases with postoperative 5‑year and 10‑year BP data is very limited, we conducted sensitivity analyses using inverse probability weighting and multiple interpolation methods for missing data to minimize bias. The results showed that the downward trends in SBP and DBP following BS were consistent with the results of the primary analysis (both *p* < 0.001, Tables S9 and S10). Both sensitivity analyses showed that the decrease in BP began as early as the first postoperative day and persisted during medium‐ and long‐term follow‐up, including at 5 and 10 years postoperatively. The magnitude of the BP‐lowering effect was generally consistent across each postoperative time points, indicating that the primary results are robust to different methods of handling missing data.

### 2.8 Effect of Bariatric Surgery on Metabolic Outcomes

Patients showed significant improvements in metabolic indicators. At 3 and 6 months, and 1, 5, and 10 years after surgery, BMI, percentage excess weight loss (EWL%), fasting blood glucose (FBG), glycosylated hemoglobin (HbA1c), fasting insulin, total cholesterol (TC), triglycerides (TGs), high‐density lipoprotein cholesterol (HDL‐C), and low‐density lipoprotein cholesterol (LDL‐C) all improved compared to pre‐surgery levels (*p* < 0.001, Table S11).

### 2.9 Mechanism Exploratory Substudy of Short‐Term Blood Pressure Reduction

In the mechanism analysis, a total of 20 patients were enrolled, and six patients dropped out due to early discharge within 6 days after BS. Among the 14 patients analyzed, the mean age was 32.5 ± 10.38 years, 64.29% of them were female, the mean BMI was 39.06 ± 4.02 kg/m^2^, and the mean SBP and DBP were 149.43 ± 9.06 mmHg and 98.36 ± 11.76 mmHg, respectively (Table S12). Within 6 days after BS, reductions in SBP and DBP were observed (Figure S2). As shown in Figure S3, ambulatory blood pressure monitoring (ABPM) revealed that on POD3, the mean daytime SBP and DBP were significantly lower than preoperative levels, and the mean nighttime SBP also decreased significantly, whereas the mean nighttime DBP showed no significant difference compared with baseline.

Table S13 shows changes in water content, urine output, and biomarkers. On Days 3 and 6, body water existed trend of decrease, intracellular water in particular decreased and 24‐h urine volume increased significantly (*p* < 0.05). In urine, absolute 24‐h sodium, chloride, and potassium excretion were both increased on POD3–6 (*p* < 0.05). At the same time, consistent with the earlier trend of decreased BP, there was a trend toward lower renin and angiotensin levels in the supine position on POD3–6, and no compensatory activation of the corresponding standing parameters or aldosterone was observed. Although not statistically significant, the trends suggest that the basal activity of the renin‐angiotensin‐aldosterone system (RAAS) may be transiently suppressed postoperatively. In addition, hormonal markers as glucagon‐like peptide 1 (GLP‐1) level also dropped (*p* < 0.05). Although our findings on the mechanisms have not passed multiple comparisons of validation, they can be used to formulate hypotheses that will guide future research into the mechanisms of early blood pressure reduction following BS.

### 2.10 Comparison Between Hypertensive and Non‐Hypertensive Patients

A total of 207 non‐hypertensive patients who met the same surgical eligibility criteria were included (Figure S4). Baseline data for non‐hypertensive patients are shown in Table S14. The average age was 35.25 ± 2.24 years. The proportion of females was 65%. The average BMI was 36.3 ± 7.56 kg/m^2^. Baseline SBP and DBP were 122.55 ± 9.38 mmHg and 77.8 ± 7.09 mmHg. Figure S5 demonstrates the comparison of BP reduction in the non‐hypertensive group and the hypertensive group. In both the short and long term, hypertensive patients had a greater drop in both SBP and DBP.

## 3 Discussion

In this study, we focused on the complete change course of BP from postoperative Day 1 to 10 years in Chinese patients coexisting with obesity and hypertension. We observed that BP declined significantly during the first 7 days postoperatively; the trend of SBP reduction began to slow down after 3 months postoperatively, while the decline in DBP began to slow after the first week postoperatively. By 1 year, BP was controlled at normal levels in 72.73% of patients, which remained stable for up to 10 years. Exploratory analyses suggested that transient suppression of the RAAS may contribute to early BP reduction independent of weight loss.

The long‐term efficacy of BS on hypertension has been confirmed by multiple studies [21]. Meta‐analysis shows that 63% of patients maintain hypertension resolution or improvement up to 5 years after BS [22, 23], and observational studies reported long‐term remission of hypertension rates of 16.4%–51% within 10–12 years postoperatively [24, 25, 26, 27, 28, 29, 30]. Although current guidelines for the management of hypertension endorse BS as an effective long‐term antihypertensive strategy, recommendations mainly target severely obese individuals (BMI ≥>37.5 kg/m^2^) [4]. At the same time, the vast majority of studies of the evidence from these guidelines came from Europe. Evidence in patients with non‐severe obesity (BMI < 37.5 kg/m^2^ [6]), who are common in Asia, remains limited. Our prospective data demonstrate that BS produced sustained BP reductions of approximately 10–15 mmHg in Chinese populations; BP control rates were slightly higher (68.3% at 5 years and 70.8% at 10 years), which may relate to the younger cohort, mean age of 36.8 years, and milder baseline hypertension (mean SBP 142.7 mmHg, DBP 93.9 mmHg). An incomplete 10‐year follow‐up may also have underestimated the prevalence.

Unlike trials such as STAMPEDE (Surgical Treatment and Medications Potentially Eradicate Diabetes Efficiently) [31], GATEWAY [1, 32], and Diabetes Surgery Study [33], where patients received adequate antihypertensive therapy, awareness and treatment rates in China are low. Most patients in our cohort were unaware of their hypertension until admission, minimizing medication effects (only 33.87% patients were using antihypertensive medicines). Furthermore, the proportion of female patients in the hypertension group was relatively low (49.6%), a discrepancy potentially reflecting the higher prevalence of hypertension among Chinese males (based on the 2021–2022 national survey, 36.8% in men vs. 26.3% in women) [12]. Despite limited mid‐ and long‐term follow‐up, these real‐world data still better reflect the impact of BS on BP, demonstrating a strong and durable antihypertensive effect for up to 10 years, even in non‐severe obesity. The observed fills an important evidence gap for Asian populations with obesity and hypertension.

We found the consistent BP reduction across participants with varying degrees of weight loss supports the hypothesis that the BS has a BP‐lowering effect that is independent of weight loss. Obesity‐related hypertension involves renal sodium retention, sympathetic activation, endothelial dysfunction, gastrointestinal hormones, and adipokines [34], and BS can modulate factors over different time layers [35, 36, 37]. Long‐term effects include decreased sympathetic activity and an increased natriuretic peptide availability [35, 36], while preoperative fasting may improve vascular endothelial function by reducing oxidative stress over weeks [38, 39]. Few studies have focused on the BP changes in the early period after BS. Prior Western reports described BP reduction within 1 or 2 weeks after BS before significant weight loss, suggesting a weight‐independent mechanism. Ahmed et al. reported a reduction in SBP of 9 mmHg was observed within 1 week after RYGB in morbidly obese patients [13, 40], and Hawkins et al. found that an average peak reduction in mean arterial pressure (MAP) of 6.25 mmHg at 2 weeks after BS [15, 41]. Pedersen et al. directly observed a significantly lower BP on 10 days after surgery, despite weight loss emerging [14, 42]. Our continuous BP monitoring in an Asian population further confirmed an immediate BP decrease starting on postoperative Day 1.

Since the aforementioned mechanisms are unlikely to act within 7 days postoperatively, we explored drivers of the immediate BP decline. The reduction of leptin has been proposed [43]; however, in our study, we did not observe any short‐term changes in leptin and adiponectin levels within 1 week after surgery, likely due to surgical stress and glucose infusion [44, 45]. Contrary to reports of increased post‐prandial GLP‐1 improving BP [46], GLP‐1 levels decreased acutely in our study, possibly due to continued fasting during the operation. Instead, we found early increases in water and sodium excretion, decreased renal sodium reabsorption, and suppression of the RAAS within 3–6 days postoperatively, with effects including reduced renal sympathetic drive, attenuated angiotensin‐mediated vasoconstriction, and the absence of aldosterone‐mediated sodium and water retention [47]. However, the changes above previously considered long‐term mechanism [37]. In conclusion, the rapid postoperative BP decline appears to be primarily associated with a reduction in early fluid and sodium retention. Further exploration is needed to elucidate the underlying pathways.

A recent meta‐analysis reported no differences in the 3‐year remission rate of hypertension between LRYGB and LSG [48], which is consistent with our findings: although the two procedures differed in their BP‐lowering effects at specific time points within the first 7 days postoperatively, their long‐term BP‐lowering effects were similar. However, our sample size was insufficient to compare hypertension remission across patients with non‐severe and severe obesity groups for each surgical treatment. To our knowledge, no previous studies have examined BP changes stratified by BMI category or BS type. Future trials comparing short‐ and long‐term BP reduction between non‐severe and severe obesity groups and surgical procedures are needed to guide more personalized treatment strategies.

The main strength of our study is the prospective observation of the full range of BP changes from 1 day to 10 years after surgery, which fills the evidence gap in the Asian population. However, our study has limitations. First, patients who developed severe adverse events (such as major bleeding, anastomotic leak, or sepsis) within the first 7 postoperative days were excluded from the short‐term BP trajectory analysis to avoid confounding effects of acute hemodynamic fluctuations and intensive treatment interventions. Although this approach preserved internal validity for BP trend assessment, it may have introduced selection bias. Second, follow‐up was incomplete at 5 and 10 years, which may have introduced attrition bias. To evaluate the robustness of our findings, we additionally performed inverse probability weighting and multiple imputation analyses, both of which yielded results consistent with the primary analyses. In the future, there is a need to strengthen the study of the level of change in blood pressure after longer term surgery. Third, we did not further discuss postoperative hypertension medication because of the lack of long‐term follow‐up and the low adherence to antihypertensive medication in the Chinese population. Finally, the mechanistic sub‐study was exploratory in nature; however, its limited sample size, single‐center design, and inclusion of only patients undergoing LSG may restrict the robustness and generalizability of the findings.

## 4 Conclusion

BS is associated with a reduction in BP in patients with obesity combined hypertensive within 7 days after surgery. Although long‐term follow‐up data are limited, our findings suggest that this reduction may be maintained for up to 10 years. This antihypertensive effect did not differ between mild, moderate, and severe obesity; different procedures (LRYGB and LSG); and different degree of weight loss. The short‐term effect may be linked to improved water and sodium excretion. This process appears to involve the renin‐angiotensin‐aldosterone system and may work independently of weight loss.

## 5 Methods

### 5.1 Prospective Cohort Construction

This was a multicenter prospective cohort study conducted at three hospitals covering the central, northern, and southern regions of China (Figure S6). The study was approved by the ethics committees (approval No. 2010S008), and informed consent was obtained from all patients. The study adhered to the Strengthening the Reporting of Cohort, Cross‐sectional, and Case‐control Studies in Surgery (STROCSS) guidelines [49]. The study is registered on Clinicaltrials.gov. (Registration ID: NCT07183137).

From September 1, 2010, to December 31, 2019, patients scheduled to undergo BS either LRYGB or LSG were prospectively recruited from three participating centers. These two procedures accounted for over 90% of all bariatric and metabolic surgeries performed in China [50], thereby minimizing procedural heterogeneity and ensuring adequate statistical power. Based on the national Chinese guidelines for bariatric/metabolic surgery applicable at the time of clinical evaluation, patients meeting the following inclusion criteria were enrolled in the prospective cohort: (1) aged 18–65 years and (2) met the guideline‐based indications for BS and had no contraindications to BS or anesthesia at the time of operation (Table S15). Exclusion criteria included (1) request to withdraw from the study during the course of the study. All surgeries were performed according to standardized protocols.

### 5.2 Study Participants

This study enrolled patients with hypertension, defined as SBP ≥140 mmHg and/or DBP ≥90 mmHg and/or prescription of  ≥1 antihypertensive agent. Patients were excluded if they experienced any of the following within 7 days postoperatively: (1) severe adverse events, (2) fewer than two BP measurements, or (3) received additional antihypertensive interventions outside the study protocol. To investigate the potential mechanisms underlying the early postoperative reduction in BP, a mechanistic exploration study was conducted. A random sample of 20 hypertensive patients was selected for this analysis.

### 5.3 End Points

In this multicenter study, the main outcomes were short‐term (POD1‐7), intermediate‐term (postoperative months 3, 6, 12), and long‐term (postoperative years 5 and 10) BP levels, and the status of hypertension (Figure 1). Blood pressure control was defined as BP < 140/90 mmHg, irrespective of antihypertensive medication use. The secondary outcomes were long‐term BMI, EWL%, FBG, HbA1c, fasting insulin, TGs, TC, HDL‐C, and LDL‐C at months 3, 6, and 12.

### 5.4 Measurement of BP

BP was measured using a corrected electronic sphygmomanometer (Omron, HEM‐7136), and two readings were obtained at an interval of 1 min for both systolic and diastolic BP. The mean of the two readings was considered the final BP. Preoperative BP was defined as the BP measured at admission by the nurse.

Postoperative daily BP values during POD1‐7 were measured in the morning until discharge. Patients discharged within 7 days after BS will be telephoned to report averaged daily BP values from two readings taken at a 1‐min interval. All patients will be asked to attend regular clinic follow‐up visits at months 3, 6, and 12 after discharge, and the BP will be measured by nurses. At 5 and 10 years after BS, patients' self‐reported weights and mean BP values measured at 1‐min intervals were obtained by telephone follow‐up. All follow‐up data were collected up to April 30, 2025, which served as the final data cutoff for this analysis.

### 5.5 Other Data Collection

Anthropometric measurements were conducted by nurses at admission and during follow‐up visits; the measurements included height, weight, and waist circumference (WC). Weight and height were measured to the nearest 0.1 kg or 0.1 cm, with all patients wearing light clothes and no shoes. BMI was calculated as body weight in kilograms (kg) divided by the square of height in meters (m^2^). WC was measured at the navel position using a nonelastic tape measure and without applying any pressure to the body surface.

Venous blood samples were percutaneously collected from the upper extremities with the patients in a seated position. FBG, TGs, TC, HDL‐C, LDL‐C, serum creatinine (Scr), and uric acid (UA) were measured by enzymatic methods with an automated biochemical analyzer (HITACHI, 7600‐020). Serum insulin levels were determined by chemiluminescence microparticle immunoassay (i2000SR, AP, USA). HbA1c was detected with high‐performance liquid chromatography (Bio‐Rad Variant II, CA, USA).

A mechanistic exploratory study was designed to investigate potential physiological mechanisms underlying the early postoperative decline in blood pressure. In the mechanistic exploratory study, each participant underwent 24‐h ABPM on preoperative Day 1 and POD_3_ to evaluate short‐term BP changes following BS. Monitoring begins the day before surgery and continues through the third day postoperatively using a SpaceLabs model 90207 device (SpaceLabs Healthcare, Snoqualmie, WA). BP is measured taken every 20 min in the daytime and every 30 min at nighttime. The period from 8:00 a.m. to 7:00 p.m. is defined as the daytime period, and the period from 8:00 p.m. to 7:00 a.m. is defined as the nighttime period. If the valid data span is less than 22 h, the monitoring session is considered invalid. For the measurements of epinephrine, norepinephrine, dopamine, antidiuretic hormone (ADH), leptin, GLP‐1, ghrelin, peptide YY (PYY), renin, angiotensin I (Ang I), angiotensin II (Ang II), and aldosterone, supernatant fluids of patients’ fasting serum samples at POD3 and POD6 after BS were collected. For the measurement of supine parameters, blood samples were collected after the patients had been fasting, awake, and at rest in a supine position for at least 8 h. Assays were performed using the enzyme‐linked immunosorbent assay (ELISA, CUSABIO, Wuhan, China). In addition, completeness of 24‐h urine collection was assessed by confirming the predefined collection period and reviewing the recorded total urine volume. Urine volume and electrolyte profiles, including sodium (Na^+^), chloride (Cl^−^), and potassium (K^+^) concentrations, were examined by an automated urine analyzer (Sysmex, UF‐1000i & UC‐3500). The absolute 24‐h urinary electrolyte excretion is calculated as Ux (mmol/L) × 24‐h volume (L). Ux represent the concentrations of x in urine. All samples were collected prior to the intravenous infusion.

### 5.6 Statistical Analysis

This study enrolled 248 patients to ensure sufficient statistical power for detecting clinically meaningful changes in blood pressure over time following BS (the estimated sample size, as calculated using G*Power version 3.1 and required to achieve approximately 0.95 power at a two‐sided α of 5% was 178 subjects).

BP was repeatedly measured at multiple postoperative time points (POD1‐7, months 3, 6, and 12, and years 5 and 10). Longitudinal changes in SBP and DBP were analyzed separately using LMMs, with follow‐up time treated as a categorical variable and participant‐specific random intercepts to account for within‐participant correlation. Models were estimated by maximum likelihood using an identity covariance structure for the random effects and an independent residual error structure. The primary models were adjusted a priori for baseline age, sex, BMI, antihypertensive medication use, baseline SBP (or DBP for DBP models), TC, surgical procedure, study center, and calendar year of surgery. Adjusted mean changes in BP from baseline with 95% CIs were estimated for each postoperative time point. Model adequacy was assessed by examining residual normality, homoscedasticity, and the distribution of random effects.

Several sensitivity analyses were performed to evaluate the robustness of the findings. First, inverse probability‐of‐observation weighting (IPOW) was applied to account for potential bias arising from incomplete follow‐up. Stabilized weights were estimated from pooled logistic regression models incorporating baseline characteristics and previous BP measurements and were incorporated into weighted LMMs. Second, missing outcome data were handled using multiple imputation by chained equations (MICE), and the primary analyses were repeated across imputed datasets with Rubin's rules used to combine estimates.

Prespecified subgroup analyses were conducted by including interaction terms between follow‐up time and subgroup variables in the LMMs. Subgroups included baseline antihypertensive medication use, baseline BMI categories (27.5–32.5 kg/m^2^, 32.5–37.5 kg/m^2^, and >37.5 kg/m^2^), surgical procedures (LRYGB vs. LSG), baseline antihypertensive medication use (no vs. yes), and TWL% at postoperative 1 year. Overall interaction effects were assessed using Wald tests, and differences between subgroup‐specific adjusted mean BP changes at each postoperative time point were evaluated using post‐estimation contrasts.

Postoperative BMI, EWL%, FBG, HbA1c, fasting insulin, TC, TG, HDL, and LDL were compared to the preoperative levels using paired *t*‐tests. Postoperatively individual within‐subject changes of epinephrine, norepinephrine, dopamine, ADH, leptin, GLP‐1, ghrelin, and PYY at POD_3_ and POD_6_ were calculated as the postoperative biomarker concentration minus the corresponding baseline concentration for each participant. Mean paired differences with 95% CIs were calculated for normally distributed variables, whereas median paired differences with bootstrap 95% CIs were calculated for skewed variables. Paired *t* tests or Wilcoxon signed‐rank tests were used accordingly. To account for multiple biomarker comparisons, nominal *p* values were adjusted using the Benjamini‐Hochberg false discovery rate (FDR) method, and FDR‐adjusted *q* values were reported.

Continuous variables with a normal distribution are reported as the mean ± SD (standard deviation). Continuous variables with a nonnormal distribution are reported as medians and interquartile ranges (IQRs). Categorical variables are summarized as frequencies. A *p* < 0.05 was considered statistically significant. All analyses were conducted by Stata version 14.0 (Stata Corp., College Station, TX).

### 5.7 Additional Analysis

An additional analysis was conducted to evaluate the impact of BS on blood pressure among individuals with and without baseline hypertension. Changes in BP were evaluated from POD1‐7 up to 10 years after surgery, and the postoperative trajectories were compared between non‐hypertensive and hypertensive patients.

## Author Contributions

**Xiaoxiao Zhong**: methodology, formal analysis, visualization, writing – original draft. **Yaoquan Cao**: methodology, formal analysis, visualization. **Xulong Sun**: methodology, formal analysis, visualization. **Pengzhou Li**: data curation, validation. **Weizheng Li**: data curation, validation. **Hong Yuan**: supervision. **Shaihong Zhu**: supervision. **Liangping Wu**: data curation, investigation. **Hua Meng**: data curation, investigation. Hua Meng: conceptualization, writing – review and editing, funding acquisition. **Yao Lu**: conceptualization, writing – review and editing, funding acquisition. **Liyong Zhu**: conceptualization, writing – review and editing, funding acquisition. All authors have read and approved the final manuscript.

## Funding

This work was supported by Noncommunicable Chronic Diseases‐National Science and Technology Major Project (2025ZD0550500, 2025ZD0550502), National Natural Science Foundation of China (82370676, 82470445, 82170437, 81500691), and Natural Science Foundation of Hunan province (2025JJ30050, 2024JJ8118, 2018JJ3783, 2017JJ3471).

## Ethics Statement

The study was approved by the ethics committees (approval No.2010S008), and written informed consent was obtained from all participants.

## Conflicts of Interest

The authors declare no conflicts of interest.

## Supporting information




**Supporting File**: mco271097‐sup‐0001‐SuppMat.docx.

## Data Availability

The raw data supporting the conclusions of this article will be made available by the authors.

## References

[1] C. A. Schiavon , A. C. Bersch‐Ferreira , E. V. Santucci , et al., “Effects of Bariatric Surgery in Obese Patients With Hypertension: The GATEWAY Randomized Trial (Gastric Bypass to Treat Obese Patients With Steady Hypertension),” Circulation 137, no. 11 (2018): 1132–1142. 10.1161/CIRCULATIONAHA.117.032130 PMC5865494 29133606

[2] C. A. Schiavon , D. L. Bhatt , D. Ikeoka , et al., “Three‐Year Outcomes of Bariatric Surgery in Patients With Obesity and Hypertension: A Randomized Clinical Trial,” Ann Intern Med 173, no. 9 (2020): 685–693. 10.7326/M19-3781 32805133

[3] C. A. Schiavon , A. B. Cavalcanti , J. D. Oliveira , et al., “Randomized Trial of Effect of Bariatric Surgery on Blood Pressure After 5 Years,” Journal of the American College of Cardiology 83, no. 6 (2024): 637–648. 10.1016/j.jacc.2023.11.032 38325988

[4] G. Mancia , R. Kreutz , M. Brunström , et al., “2023 ESH Guidelines for the Management of Arterial Hypertension: The Task Force for the Management of Arterial Hypertension of the European Society of Hypertension: Endorsed by the International Society of Hypertension (ISH) and the European Renal Association (ERA),” Journal of Hypertension 41, no. 12 (2023): 1874–2071. 10.1097/HJH.0000000000003480 37345492

[5] J. G. Wang , “Chinese Guidelines for the Prevention and Treatment of Hypertension (2024 revision),” Journal of Geriatric Cardiology 22, no. 1 (2025): 1–149. 10.26599/1671-5411.2025.01.008 PMC11937835 40151633

[6] Committee NCPGoOME . National Clinical Practice Guideline on Obesity Management Editorial Committee. Guideline for Diagnosis and Treatment of Obesity (2024 edition), Chinese Circulation Journal 2025;40(01):6–30.

[7] K. C. Koskinas , E. M. Van Craenenbroeck , C. Antoniades , et al., “Obesity and Cardiovascular Disease: An ESC Clinical Consensus Statement,” European Journal of Preventive Cardiology 32, no. 3 (2025): 184–220. 10.1093/eurjpc/zwae279 39210708

[8] I. J. Neeland , P. Poirier , and J. P. Despres , “Cardiovascular and Metabolic Heterogeneity of Obesity Clinical Challenges and Implications for Management,” Circulation 137, no. 13 (2018): 1391–1406. 10.1161/CIRCULATIONAHA.117.029617 PMC5875734 29581366

[9] M. E. Piche , P. Poirier , I. Lemieux , and J. P. Despres , “Overview of Epidemiology and Contribution of Obesity and Body Fat Distribution to Cardiovascular Disease: An Update,” Progress in Cardiovascular Diseases 61, no. 2 (2018): 103–113. 10.1016/j.pcad.2018.06.004 29964067

[10] Z. Wang , Z. Chen , L. Zhang , et al., “Status of Hypertension in China: Results From the China Hypertension Survey, 2012–2015,” Circulation 137, no. 22 (2018): 2344–2356. 10.1161/CIRCULATIONAHA.117.032380 29449338

[11] X. Sun , A. F. Yan , Z. Shi , et al., “Health Consequences of Obesity and Projected Future Obesity Health Burden in China,” Obesity (Silver Spring) 30, no. 9 (2022): 1724–1751. 10.1002/oby.23472 36000246

[12] X. Cao , X. Wang , Y. Tian , et al., “Trends and Sociodemographic Patterns in Hypertension Prevalence and Treatment in China,” Med 6, no. 11 (2025): 100808. 10.1016/j.medj.2025.100808 40865522

[13] A. R. Ahmed , G. Rickards , D. Coniglio , et al., “Laparoscopic Roux‐en‐Y Gastric Bypass and Its Early Effect on Blood Pressure,” Obesity Surgery 19, no. 7 (2009): 845–849. 10.1007/s11695-008-9671-z 18758869

[14] J. S. Pedersen , C. Borup , M. Damgaard , et al., “Early 24‐Hour Blood Pressure Response to Roux‐en‐Y Gastric Bypass in Obese Patients,” Scandinavian Journal of Clinical and Laboratory Investigation 77, no. 1 (2017): 53–59. 10.1080/00365513.2016.1258725 27905219

[15] D. N. Hawkins , B. J. Faler , Y. U. Choi , and B. M. Prasad , “Time Course of Blood Pressure Decrease After Bariatric Surgery in Normotensive and Hypertensive Patients,” Obesity Surgery 28, no. 7 (2018): 1845–1851. 10.1007/s11695-017-3091-x 29725978

[16] R. Samson , G. Milligan , E. Lewine , et al., “Effect of Sleeve Gastrectomy on Hypertension,” Journal of the American Society of Hypertension 12, no. 11 (2018): e19–e25. 10.1016/j.jash.2018.09.007 30322789

[17] J. M. Isbell , R. A. Tamboli , E. N. Hansen , et al., “The Importance of Caloric Restriction in the Early Improvements in Insulin Sensitivity After Roux‐en‐Y Gastric Bypass Surgery,” Diabetes Care 33, no. 7 (2010): 1438–1442. 10.2337/dc09-2107 PMC2890335 20368410

[18] D. Elahi , P. Galiatsatos , A. Rabiee , et al., “Mechanisms of Type 2 Diabetes Resolution After Roux‐en‐Y Gastric Bypass,” Surgery for Obesity and Related Diseases 10, no. 6 (2014): 1028–1039. 10.1016/j.soard.2014.07.011 PMC10131082 25443077

[19] T. Xu , C. Wang , H. Zhang , et al., “Timing of Maximal Weight Reduction Following Bariatric Surgery: A Study in Chinese Patients,” Frontiers in Endocrinology 11 (2020): 615. 10.3389/fendo.2020.00615 PMC7522214 33042006

[20] L. Ribeiro‐Parenti , C. Baratte , and T. Poghosyan , “Weight Regain After Bariatric Surgery,” Journal of Clinical Medicine 12, no. 9 (2023): 3265. 10.3390/jcm12093265 PMC10179663 37176705

[21] C. A. Schiavon , A. Pio‐Abreu , and L. F. Drager , “Bariatric Surgery for Resistant Hypertension: Working in Progress!,” Current Hypertension Reports 22, no. 8 (2020): 55. 10.1007/s11906-020-01055-z 32671558

[22] H. Buchwald , Y. Avidor , E. Braunwald , et al., “Bariatric Surgery: A Systematic Review and Meta‐Analysis. Review,” JAMA 292, no. 14 (2004): 1724–1737. 10.1001/jama.292.14.1724 15479938

[23] A. R. Vest , H. M. Heneghan , S. Agarwal , P. R. Schauer , and J. B. Young , “Bariatric Surgery and Cardiovascular Outcomes: A Systematic Review,” Review Heart 98, no. 24 (2012): 1763–1777. 10.1136/heartjnl-2012-301778 23077152

[24] L. Sjöström , A. K. Lindroos , M. Peltonen , et al., “Lifestyle, Diabetes, and Cardiovascular Risk Factors 10 Years After Bariatric Surgery,” New England Journal of Medicine 351, no. 26 (2004): 2683–2693. 10.1056/NEJMoa035622 15616203

[25] A. Heald , F. Mannan , R. Wiltshire , et al., “The Effects of Bariatric Surgery on the Requirement for Antihypertensive Treatment in Type 2 Diabetes: Insights From a Long‐Term Follow‐Up Study,” Diabetes Therapy 15, no. 9 (2024): 2097–2107. 10.1007/s13300-024-01627-1 PMC11330420 39078476

[26] P. Hallersund , L. Sjöström , T. Olbers , et al., “Gastric Bypass Surgery Is Followed by Lowered Blood Pressure and Increased Diuresis—Long Term Results From the Swedish Obese Subjects (SOS) Study,” PLoS ONE 7, no. 11 (2012): e49696. 10.1371/journal.pone.0049696 PMC3510228 23209592

[27] T. D. Adams , L. E. Davidson , S. E. Litwin , et al., “Weight and Metabolic Outcomes 12 Years After Gastric Bypass,” New England Journal of Medicine 377, no. 12 (2017): 1143–1155. 10.1056/NEJMoa1700459 PMC5737957 28930514

[28] L. Sjostrom , A. K. Lindroos , M. Peltonen , et al., “Lifestyle, Diabetes, and Cardiovascular Risk Factors 10 Years After Bariatric Surgery,” New England Journal of Medicine 351, no. 26 (2004): 2683–2693. 10.1056/NEJMoa035622 15616203

[29] T. D. Adams , L. E. Davidson , S. E. Litwin , et al., “Weight and Metabolic Outcomes 12 Years After Gastric Bypass,” New England Journal of Medicine 377, no. 12 (2017): 1143–1155. 10.1056/NEJMoa1700459 PMC5737957 28930514

[30] P. Hallersund , L. Sjostrom , T. Olbers , et al., “Gastric Bypass Surgery Is Followed by Lowered Blood Pressure and Increased Diuresis—Long Term Results from the Swedish Obese Subjects (SOS) Study Article,” PloS One 7, no. 11 (2012): e49696. 10.1371/journal.pone.0049696 PMC3510228 23209592

[31] P. R. Schauer , D. L. Bhatt , J. P. Kirwan , et al., “Bariatric Surgery Versus Intensive Medical Therapy for Diabetes‐5‐Year Outcomes,” New England Journal of Medicine 376, no. 7 (2017): 641–651. 10.1056/NEJMoa1600869 PMC5451258 28199805

[32] C. A. Schiavon , A. C. Bersch‐Ferreira , E. V. Santucci , et al., “Effects of Bariatric Surgery in Obese Patients With Hypertension: The GATEWAY Randomized Trial (Gastric Bypass to Treat Obese Patients With Steady Hypertension),” Circulation 137, no. 11 (2018): 1132–1142. 10.1161/CIRCULATIONAHA.117.032130 PMC5865494 29133606

[33] S. Ikramuddin , J. Korner , W. J. Lee , et al., “Lifestyle Intervention and Medical Management With vs Without Roux‐en‐Y Gastric Bypass and Control of Hemoglobin A(1c), LDL Cholesterol, and Systolic Blood Pressure at 5 Years in the Diabetes Surgery Study,” JAMA 319, no. 3 (2018): 266–278. 10.1001/jama.2017.20813 PMC5833547 29340678

[34] J. E. Hall , J. M. do Carmo , A. A. da Silva , Z. Wang , and M. E. Hall , “Obesity, Kidney Dysfunction and Hypertension: Mechanistic Links,” Nature Reviews Nephrology 15, no. 6 (2019): 367–385. 10.1038/s41581-019-0145-4 PMC7278043 31015582

[35] G. Seravalle , M. Colombo , P. Perego , et al., “Long‐Term Sympathoinhibitory Effects of Surgically Induced Weight Loss in Severe Obese Patients,” Hypertension 64, no. 2 (2014): 431–437. 10.1161/HYPERTENSIONAHA.113.02988 24866140

[36] P. K. Bonfils , M. Taskiran , M. Damgaard , et al., “Roux‐en‐Y Gastric Bypass Alleviates Hypertension and Is Associated With an Increase in Mid‐Regional Pro‐Atrial Natriuretic Peptide in Morbid Obese Patients,” Journal of Hypertension 33, no. 6 (2015): 1215–1225. 10.1097/HJH.0000000000000526 25668345

[37] R. Samson , K. Ayinapudi , T. H. Le Jemtel , and S. Oparil , “Obesity, Hypertension, and Bariatric Surgery,” Current Hypertension Reports 22, no. 7 (2020): 46. 10.1007/s11906-020-01049-x 32591918

[38] R. Nicoll and M. Y. Henein , “Caloric Restriction and Its Effect on Blood Pressure, Heart Rate Variability and Arterial Stiffness and Dilatation: A Review of the Evidence,” International Journal of Molecular Sciences 19, no. 3 (2018): 751. 10.3390/ijms19030751 PMC5877612 29518898

[39] H. Zhang , Y. Pu , J. Chen , et al., “Gastrointestinal Intervention Ameliorates High Blood Pressure Through Antagonizing Overdrive of the Sympathetic Nerve in Hypertensive Patients and Rats,” Journal of the American Heart Association 3, no. 5 (2014): e000929. 10.1161/JAHA.114.000929 PMC4323786 25240055

[40] A. R. Ahmed , G. Rickards , D. Coniglio , et al., “Laparoscopic Roux‐en‐Y Gastric Bypass and Its Early Effect on Blood Pressure,” Obesity Surgery 19, no. 7 (2009): 845–849. 10.1007/s11695-008-9671-z 18758869

[41] D. N. Hawkins , B. J. Faler , Y. U. Choi , and B. M. Prasad , “Time Course of Blood Pressure Decrease After Bariatric Surgery in Normotensive and Hypertensive Patients,” Obesity Surgery 28, no. 7 (2018): 1845–1851. 10.1007/s11695-017-3091-x 29725978

[42] J. S. Pedersen , C. Borup , M. Damgaard , et al., “Early 24‐hour Blood Pressure Response to Roux‐en‐Y Gastric Bypass in Obese Patients,” Scandinavian Journal of Clinical & Laboratory Investigation 77, no. 1 (2017): 53–59. 10.1080/00365513.2016.1258725 27905219

[43] R. Samson , K. Ayinapudi , T. H. Le Jemtel , and S. Oparil , “Obesity, Hypertension, and Bariatric Surgery,” Current Hypertension Reports 22, no. 7 (2020): 46. 10.1007/s11906-020-01049-x 32591918

[44] J. W. Kolaczynski , R. V. Considine , J. Ohannesian , et al., “Responses of Leptin to Short‐Term Fasting and Refeeding in Humans: A Link With Ketogenesis but Not Ketones Themselves,” Diabetes 45, no. 11 (1996): 1511–1515. 10.2337/diab.45.11.1511 8866554

[45] J. W. Stephens , T. Min , G. Dunseath , R. Churm , J. D. Barry , and S. L. Prior , “Temporal Effects of Laparoscopic Sleeve Gastrectomy on Adipokines, Inflammation, and Oxidative Stress in Patients With Impaired Glucose Homeostasis,” Surgery for Obesity and Related Diseases 15, no. 12 (2019): 2011–2017. 10.1016/j.soard.2019.04.006 31711947

[46] P. Larraufie , G. P. Roberts , A. K. McGavigan , et al., “Important Role of the GLP‐1 Axis for Glucose Homeostasis After Bariatric Surgery,” Cell Reports 26, no. 6 (2019): 1399–1408.e6. 10.1016/j.celrep.2019.01.047 PMC6367566 30726726

[47] M. Berney , N. Vakilzadeh , M. Maillard , et al., “Bariatric Surgery Induces a Differential Effect on Plasma Aldosterone in Comparison to Dietary Advice Alone,” Frontiers in Endocrinology 12 (2021): 745045. 10.3389/fendo.2021.745045 PMC8525894 34675881

[48] Y. Lee , A. G. Doumouras , J. Yu , et al., “Laparoscopic Sleeve Gastrectomy Versus Laparoscopic Roux‐en‐Y Gastric Bypass: A Systematic Review and Meta‐Analysis of Weight Loss, Comorbidities, and Biochemical Outcomes From Randomized Controlled Trials,” Annals of Surgery 273, no. 1 (2021): 66–74. 10.1097/SLA.0000000000003671 31693504

[49] R. Rashid , C. Sohrabi , A. Kerwan , et al., “The STROCSS 2024 guideline: strengthening the reporting of cohort, cross‐sectional, and case–control studies in surgery,” International Journal of Surgery 110, no. 6 (2024), 3151–3165. 10.1097/JS9.0000000000001268 PMC11175759 38445501

[50] “Greater China Bariatric & Metabolic Surgery Database,” Chinese Journal of Practical Surgery 45. no. 05 (2025): 538–552.

