# Supplementary material for: Short‐ and Long‐Term Control of Hypertension After Bariatric Surgery: A 10‐Year Prospective Study
Source: MedComm (2020). 2026 Oct 29;7(11):e71097. doi: 10.1002/mco2.71097 (PMC13717295; doi:10.1002/mco2.71097)
Supplement: Supplementary file 1 [file MCO2-7-e71097-s001.docx]

**Short- and Long-Term Control of Hypertension After Bariatric Surgery: A 10-Year Prospective Study**

**Running title: Bariatric Surgery and Hypertension**

Xiaoxiao Zhong, et al.

Corresponding Author:

Liyong Zhu, MD, PhD, the Third Xiangya Hospital of Central South University, Changsha, 410013, China.

E-mail address: [zly8128@csu.edu.cn](mailto:zly8128@csu.edu.cn) (Liyong Zhu)

Yao Lu, MD, PhD, the Third Xiangya Hospital of Central South University, Changsha, 410013, China.

E-mail address: [luyao0719@163.com](mailto:luyao0719@163.com) (Yao Lu)

Hua Meng, MD, PhD, the China-Japan Friendship Hospital, Beijing, China.

E-mail address: [menghuade@hotmail.com](mailto:menghuade@hotmail.com) (Hua Meng)

**This file includes:**

Supplemental Figures S1-S6

Supplemental Tables S1-S15

**Supplemental Figures**

**Figure S1** Flowchart of prospective cohort construction.

**Figure S2** Blood pressure changes after BS in patients of the mechanism analysis. SBP, systolic blood pressure; DBP, diastolic blood pressure.

**Figure S3** Changes in 24-hour ambulatory blood pressure on preoperative and postoperative day 3 in the mechanistic analysis. A, Twenty-four-hour SBP on preoperative and POD3; B, Daytime SBP on preoperative and POD3; C, Nighttime SBP on preoperative and POD3; D, Twenty-four-hour DBP on preoperative and POD3; E, Daytime DBP on preoperative and POD3; F, Nighttime DBP on preoperative and POD3. SBP, systolic blood pressure; DBP, diastolic blood pressure; POD3, postoperative day 3. *P<0.05, **P<0.001, ***P<0.001, ****P<0.0001, compared between preoperative and POD3.

**Figure S4** Flowchart of enrolled patients in additional analysis.

**Figure S5** The trajectory of the change of blood pressure in the long and short term after bariatric surgery by preoperative status of hypertension. A, change of SBP from POD_1-7_ to postoperative 10 years by preoperative status of hypertension; B, change of DBP from POD_1-7_ to postoperative 10 years by preoperative status of hypertension; C, change of SBP from postoperative days 1 to 7 by preoperative status of hypertension; D, change of DBP from postoperative days 1 to 7 by preoperative status of hypertension. SBP, systolic blood pressure; DBP, diastolic blood pressure; POD_1-7_, postoperative days 1 to 7.

*P<0.05, **P<0.001, ***P<0.001, ****P<0.0001, compared between hypertension and non-hypertension patients.

**Figure S6** Distribution of enrolled patients in the study. This map was created based on a standard map with review number GS(2019)1651, downloaded from the standard map service from the Ministry of Natural Resources of P.R.China.

**Supplemental Tables**

Table S1. Numbers of eligible participants, participants with blood pressure assessment, and lost to follow-up at each follow-up time point.

| **Follow-up time** | **Eligible Participants (n)** | **Assessed Participants(n)** | **Lost to follow-up (n)** |
| --- | --- | --- | --- |
| Baseline | 248 | 248 | 0 |
| 1-7 Days | 248 | 246 | 2 |
| 3 months | 248 | 83 | 165 |
| 6 months | 248 | 37 | 211 |
| 1 year | 248 | 33 | 215 |
| 5 years | 248 | 41 | 207 |
| 10 years | 32 | 24 | 8 |

Table S2 Long-term blood pressure control following bariatric surgery.

| Time | Patients assessed (n) | SBP, mmHg | DBP, mmHg | BP controlled <140/90 mmHg (%), n/N |
| --- | --- | --- | --- | --- |
| Baseline | 248 | 142.7 ± 13.96 | 93.93 ± 10.94 | 14.92, 37/248 |
| 1-7 Days | 246 | 134.23 ± 10.89^****^ | 83.75 ± 7.99^****^ | 62.90^****^, 156/248 |
| 3 Months | 83 | 126.66 ± 14.57^****^ | 83.37 ± 10.25^****^ | 73.49^****^, 61/83 |
| 6 Months | 37 | 124.54 ± 16.59^****^ | 82.28 ± 12.14^****^ | 61.11^****^, 22/36 |
| 1 Years | 33 | 126.15 ± 17.74^****^ | 82.38 ± 10.06^****^ | 72.73^****^, 24/33 |
| 5 Years | 41 | 123.76 ± 15.83^****^ | 87.17 ± 8.05^***^ | 68.29^****^, 28/41 |
| 10 Years | 24 | 124.83 ± 13.08^****^ | 81.58 ± 9.93^****^ | 70.83^****^, 17/24 |

BMI, body mass index; SBP, systolic blood pressure; DBP, diastolic blood pressure.

^*^P<0.05, ^**^P<0.01, ^***^P<0.001, ^****^P<0.0001, compared with Baseline.

^#^P<0.05, compared with 6 Months.

Table S3 Short-term blood pressure control following bariatric surgery.

| Time | SBP, mmHg | DBP, mmHg | BP controlled <140/90 mmHg (%), n/N |
| --- | --- | --- | --- |
| Baseline | 142.7 ± 13.96 | 93.93 ± 10.94 | 14.92, 37/248 |
| POD_1_ | 134.2 ± 15.02^****^ | 83.04 ± 11.78^****^ | 56.91, 140/246 |
| POD_2_ | 135.99 ± 13.79^****^ | 84.88 ± 10.22^****^ | 52.07, 113/217 |
| POD_3_ | 134.87 ± 13.71^****^ | 84.23 ± 9.88^****^ | 55.43, 102/184 |
| POD_4_ | 133 ± 13.26^****^ | 83.58 ± 10.18^****^ | 60.57, 106/175 |
| POD_5_ | 131.04 ± 13.33^****^ | 82.91 ± 10.82^****^ | 61.84, 83/135 |
| POD_6_ | 131.19 ± 13.77^****^ | 83.96 ± 11.28^****^ | 58.59, 58/99 |
| POD_7_ | 129.15 ± 12.1^****^ | 83.15 ± 9^****^ | 64.18, 43/67 |

SBP, systolic blood pressure; DBP, diastolic blood pressure.

^****^P<0.0001, compared with Baseline.

^#^P<0.05, compared with POD_1_.

Table S4 Longitudinal changes in blood pressure according to baseline BMI categories.

| Time | Adjusted SBP change, mmHg (95% CI) | | |  | Adjusted DBP change, mmHg (95% CI) | | |  |
| --- | --- | --- | --- | --- | --- | --- | --- | --- |
|  | 27.5-32.5 kg/m^2^ (n=69) | 32.5-37.5 kg/m^2^ (n=60) | > 37.5 kg/m^2^ (n=119) | P value | 27.5-32.5 kg/m^2^ (n=69) | 32.5-37.5 kg/m^2^ (n=60) | > 37.5 kg/m^2^ (n=119) | P value |
| POD1 | -9.33 (-12.86, -5.80) | -8.98 (-12.82, -5.15) | -7.95 (-10.68, -5.22) | 0.260 | -9.70 (-12.35, -7.04) | -11.00 (-13.88, -8.12) | -11.82 (-13.88, -9.77) | 0.965 |
| POD2 | -5.57 (-9.19, -1.94) | -6.35 (-10.38, -2.33) | -8.22 (-11.05, -5.38) | 0.601 | -7.60 (-10.33, -4.87) | -9.28 (-12.31, -6.25) | -9.24 (-11.37, -7.11) | 0.762 |
| POD3 | -5.53 (-9.20, -1.87) | -8.71 (-12.96, -4.46) | -11.14 (-14.17, -8.10) | 0.056 | -8.81 (-11.57, -6.06) | -11.67 (-14.87, -8.47) | -9.72 (-12.01, -7.44) | 0.218 |
| POD4 | -9.50 (-13.30, -5.70) | -10.42 (-14.82, -6.02) | -10.69 (-13.71, -7.66) | 0.683 | -9.55 (-12.41, -6.70) | -12.62 (-15.93, -9.31) | -10.28 (-12.56, -8.01) | 0.157 |
| POD5 | -11.00 (-15.24, -6.76) | -12.55 (-17.45, -7.64) | -13.73 (-16.95, -10.51) | 0.611 | -9.81 (-12.99, -6.62) | -14.03 (-17.72, -10.34) | -12.77 (-15.19, -10.35) | 0.432 |
| POD6 | -12.37 (-17.04, -7.71) | -14.43 (-20.02, -8.84) | -12.03 (-15.63, -8.44) | 0.368 | -10.33 (-13.84, -6.82) | -12.18 (-16.39, -7.97) | -10.32 (-13.02, -7.61) | 0.333 |
| POD7 | -12.95 (-18.13, -7.77) | -16.46 (-23.74, -9.17) | -15.06 (-19.24, -10.88) | 0.552 | -11.31 (-15.21, -7.41) | -12.60 (-18.08, -7.12) | -11.59 (-14.73, -8.44) | 0.603 |
| 1-7 Days | -7.66 (-11.17, -4.14) | -8.70 (-12.53, -4.86) | -9.16 (-11.88, -6.44) | 0.580 | -9.21 (-11.85, -6.57) | -11.51 (-14.40, -8.63) | -10.62 (-12.67, -8.58) | 0.429 |
| 3 months | -15.54 (-20.63, -10.44) | -15.28 (-21.13, -9.44) | -16.67 (-20.59, -12.74) | 0.969 | -11.08 (-14.91, -7.24) | -9.10 (-13.50, -4.70) | -11.96 (-14.91, -9.01) | 0.441 |
| 6 months | -12.29 (-19.52, -5.05) | -14.11 (-20.89, -7.33) | -17.44 (-23.50, -11.38) | 0.642 | -8.73 (-14.18, -3.28) | -10.34 (-15.64, -5.04) | -7.40 (-11.97, -2.84) | 0.416 |
| 1 year | -7.04 (-14.61, 0.54) | -14.22 (-20.78, -7.66) | -21.32 (-28.40, -14.24) | 0.032 | -8.80 (-14.50, -3.10) | -9.19 (-14.13, -4.25) | -9.10 (-14.19, -4.00) | 0.843 |
| 5 years | -12.51 (-21.77, -3.25) | -17.68 (-24.47, -10.88) | -20.64 (-25.53, -15.75) | 0.359 | -4.60 (-11.57, 2.38) | -6.89 (-12.01, -1.78) | -12.00 (-15.68, -8.32) | 0.259 |
| 10 years | -13.93 (-22.41, -5.45) | -12.26 (-21.47, -3.05) | -14.88 (-21.94, -7.81) | 0.993 | -7.81 (-14.19, -1.42) | -11.85 (-18.78, -4.91) | -12.73 (-18.04, -7.41) | 0.816 |
|  |  |  |  | P for interaction = 0.736 |  |  |  | P for interaction = 0.912 |

Change over time in blood pressure post bariatric surgery by baseline BMI category were estimated using maximum-likelihood linear mixed-effects models with participant-level random intercepts. Models were adjusted for age, sex, baseline antihypertensive medication use, the corresponding baseline BP, baseline total cholesterol, study center, surgical procedure, and calendar year of surgery. The interaction term (time × BMI category) was tested using Wald tests. SBP, systolic blood pressure; DBP, diastolic blood pressure; 95%CI = change from baseline 95% confidence interval.

Table S5 Longitudinal changes in blood pressure according to surgical procedures.

| Time | Adjusted SBP change, mmHg (95% CI) | | | Adjusted DBP change, mmHg (95% CI) | | |
| --- | --- | --- | --- | --- | --- | --- |
|  | LRYBG (n=99) | LSG (n=149) | P value | LRYBG (n=99) | LSG (n=149) | P value |
| POD1 | -12.73 (-15.68, -9.77) | -5.78 (-8.22, -3.35) | <0.001 | -9.83 (-12.06, -7.61) | -11.83 (-13.66, -9.99) | 0.120 |
| POD2 | -8.33 (-11.29, -5.37) | -6.16 (-8.75, -3.56) | 0.271 | -6.81 (-9.04, -4.58) | -10.20 (-12.15, -8.24) | 0.571 |
| POD3 | -9.42 (-12.39, -6.45) | -8.72 (-11.57, -5.87) | 0.752 | -7.58 (-9.82, -5.34) | -11.92 (-14.07, -9.78) | 0.935 |
| POD4 | -11.50 (-14.58, -8.42) | -9.57 (-12.41, -6.73) | 0.361 | -8.51 (-10.82, -6.19) | -12.27 (-14.41, -10.13) | 0.768 |
| POD5 | -15.39 (-18.77, -12.00) | -10.62 (-13.69, -7.56) | 0.035 | -11.39 (-13.94, -8.83) | -12.57 (-14.88, -10.26) | 0.075 |
| POD6 | -12.52 (-16.46, -8.57) | -12.70 (-16.00, -9.40) | 0.924 | -10.69 (-13.66, -7.71) | -10.72 (-13.20, -8.23) | 0.030 |
| POD7 | -14.56 (-19.30, -9.82) | -14.49 (-18.27, -10.70) | 0.998 | -11.84 (-15.42, -8.27) | -11.79 (-14.64, -8.93) | 0.062 |
| 1-7 Days | -10.73 (-13.68, -7.79) | -7.18 (-9.61, -4.75) | 0.060 | -8.79 (-11.01, -6.57) | -11.55 (-13.38, -9.72) | 0.307 |
| 3 months | -14.91 (-21.02, -8.81) | -15.51 (-18.65, -12.38) | 0.845 | -8.32 (-12.92, -3.71) | -12.31 (-14.68, -9.94) | 0.930 |
| 6 months | -13.70 (-20.53, -6.86) | -15.04 (-19.66, -10.42) | 0.732 | -7.57 (-12.72, -2.41) | -9.47 (-13.02, -5.92) | 0.460 |
| 1 year | -9.93 (-16.76, -3.10) | -16.69 (-21.69, -11.69) | 0.106 | -7.67 (-12.61, -2.72) | -9.99 (-13.76, -6.21) | 0.542 |
| 5 years | -8.79 (-30.84, 13.26) | -17.63 (-21.45, -13.82) | 0.433 | -6.21 (-22.88, 10.46) | -10.21 (-13.08, -7.33) | 0.979 |
| 10 years | -11.83 (-24.39, 0.73) | -13.61 (-18.72, -8.50) | 0.788 | -2.85 (-12.32, 6.62) | -12.91 (-16.76, -9.06) | 0.259 |
|  |  |  | P for interaction = 0.012 |  |  | P for interaction = 0.194 |

Change over time in blood pressure post bariatric surgery by surgical procedures were estimated using maximum-likelihood linear mixed-effects models with participant-level random intercepts. Models were adjusted for age, sex, baseline BMI, baseline antihypertensive medication use, the corresponding baseline BP, baseline total cholesterol, study center, and calendar year of surgery. The interaction term (time × surgical procedure) was tested using Wald tests. BMI, body mass index; SBP, systolic blood pressure; DBP, diastolic blood pressure. LRYBG, laparoscopic Roux-en-Y geriatric bypass; LSG, laparoscopic sleeve gastrectomy; 95%CI = change from baseline 95% confidence interval.
Table S6 Longitudinal changes in blood pressure according to antihypertensive medication use.

| Time | Adjusted SBP change, mmHg (95% CI) | | | Adjusted DBP change, mmHg (95% CI) | | |
| --- | --- | --- | --- | --- | --- | --- |
|  | No antihypertensive medication (n=164) | Antihypertensive medication (n=84) | P value | No antihypertensive medication (n=164) | Antihypertensive medication (n=84) | P value |
| POD1 | -10.77 (-13.07, -8.46) | -4.33 (-7.55, -1.10) | 0.001 | -14.03 (-15.75, -12.31) | -5.13 (-7.54, -2.73) | <0.001 |
| POD2 | -10.03 (-12.44, -7.61) | -1.29 (-4.59, 2.01) | <0.001 | -11.38 (-13.17, -9.58) | -3.75 (-6.21, -1.29) | 0.003 |
| POD3 | -11.27 (-13.85, -8.69) | -4.14 (-7.49, -0.79) | 0.004 | -12.12 (-14.05, -10.20) | -5.61 (-8.10, -3.11) | <0.001 |
| POD4 | -12.13 (-14.73, -9.53) | -6.61 (-10.07, -3.15) | 0.048 | -12.70 (-14.64, -10.76) | -6.45 (-9.03, -3.87) | <0.001 |
| POD5 | -14.80 (-17.61, -11.98) | -8.68 (-12.50, -4.86) | 0.042 | -14.76 (-16.86, -12.67) | -7.21 (-10.06, -4.36) | <0.001 |
| POD6 | -14.62 (-17.73, -11.51) | -8.73 (-13.07, -4.40) | 0.095 | -13.08 (-15.40, -10.76) | -6.11 (-9.35, -2.88) | 0.001 |
| POD7 | -15.34 (-18.98, -11.71) | -13.06 (-18.15, -7.98) | 0.851 | -13.71 (-16.42, -11.00) | -7.85 (-11.64, -4.06) | 0.014 |
| 1-7 Days | -11.09 (-13.40, -8.79) | -3.79 (-7.01, -0.58) | 0.001 | -13.10 (-14.82, -11.39) | -5.22 (-7.62, -2.83) | 0.002 |
| 3 months | -19.30 (-22.65, -15.96) | -9.42 (-14.17, -4.67) | 0.003 | -14.87 (-17.36, -12.37) | -3.72 (-7.27, -0.18) | <0.001 |
| 6 months | -18.06 (-22.82, -13.30) | -8.91 (-15.29, -2.54) | 0.059 | -11.53 (-15.08, -7.98) | -3.19 (-8.11, 1.74) | 0.135 |
| 1 year | -17.42 (-22.50, -12.34) | -9.24 (-15.85, -2.64) | 0.117 | -12.54 (-16.33, -8.75) | -2.69 (-7.44, 2.07) | 0.046 |
| 5 years | -21.34 (-25.33, -17.34) | -9.05 (-18.16, 0.06) | 0.032 | -11.33 (-14.31, -8.34) | -6.02 (-12.82, 0.79) | 0.689 |
| 10 years | -16.36 (-21.97, -10.74) | -9.34 (-17.75, -0.92) | 0.293 | -14.85 (-19.04, -10.66) | -3.10 (-9.38, 3.18) | 0.036 |
|  |  |  | P for interaction = 0.006 |  |  | P for interaction <0.001 |

Change over time in blood pressure post bariatric surgery by baseline BMI category were estimated using maximum-likelihood linear mixed-effects models with participant-level random intercepts. Models were adjusted for age, sex, baseline BMI, the corresponding baseline BP, baseline total cholesterol, study center, surgical procedure, and calendar year of surgery. The interaction term (time × antihypertensive medication use) was tested using Wald tests. SBP, systolic blood pressure; DBP, diastolic blood pressure; 95%CI = change from baseline 95% confidence interval.

Table S7. Association of time-varying total weight loss percentage (TWL%) with longitudinal changes in blood pressure after bariatric surgery.

| Time | Adjusted SBP change, mmHg (95% CI) | P value | Adjusted DBP change, (95% CI), mmHg | P value |
| --- | --- | --- | --- | --- |
| 3 Months | -16.29 (-19.35, -13.24) | <0.001 | -12.71 (-14.72, -10.71) | <0.001 |
| 6 Months | -14.55 (-18.84, -10.26) | <0.001 | -8.84 (-11.74, -5.95) | <0.001 |
| 1 Years | -14.71 (-19.29, -10.13) | <0.001 | -9.94 (-12.96, -6.93) | <0.001 |
| 5 Years | -17.62 (-22.07, -13.16) | <0.001 | -10.89 (-13.86, -7.92) | <0.001 |
| 10 Years | -15.79 (-21.28, -10.31) | <0.001 | -14.47 (-18.18, -10.77) | <0.001 |
|  |  | P for interaction = 0.466 |  | P for interaction = 0.102 |
| Time-varying TWL% (per 1% increase) | -0.22 (-0.48, 0.05) | 0.105 | 0.00 (-0.17, 0.17) | 0.998 |

Change in blood pressure over time in the postoperative period following bariatric surgery were estimated using maximum-likelihood linear mixed-effects models with participant-level random intercepts. Models were adjusted for age, sex, baseline BMI, baseline antihypertensive medication use, the corresponding baseline BP, baseline total cholesterol, study center, surgical procedure, calendar year of surgery, and TWL% as a time-varying covariate. The interaction term (time × TWL%) was tested using Wald tests. β coefficients for TWL% represent the estimated blood pressure change associated with each 1% increase in TWL%.

Table S8 Longitudinal changes in blood pressure according to postoperative 1-year TWL% groups.

| Time | Adjusted SBP change, mmHg (95% CI) | | | Adjusted DBP change, mmHg (95% CI) | | |
| --- | --- | --- | --- | --- | --- | --- |
|  | Lower TWL% (n=34) | Higher TWL% (n=34) | P value | Lower TWL% (n=34) | Higher TWL% (n=34) | P value |
| POD1 | -10.12 (-15.22, -5.02) | -9.47 (-14.53, -4.41) | 0.859 | -10.73 (-14.37, -7.10) | -8.06 (-11.66, -4.45) | 0.306 |
| POD2 | -10.30 (-15.40, -5.20) | -12.38 (-17.44, -7.32) | 0.57 | -7.91 (-11.55, -4.28) | -10.79 (-14.40, -7.19) | 0.27 |
| POD3 | -10.02 (-15.16, -4.88) | -13.75 (-19.30, -8.20) | 0.334 | -9.66 (-13.33, -5.99) | -11.09 (-15.04, -7.13) | 0.603 |
| POD4 | -14.13 (-19.42, -8.83) | -12.12 (-17.82, -6.42) | 0.613 | -11.67 (-15.45, -7.90) | -10.26 (-14.32, -6.19) | 0.616 |
| POD5 | -18.60 (-24.55, -12.64) | -18.24 (-24.68, -11.80) | 0.936 | -12.65 (-16.90, -8.40) | -11.65 (-16.25, -7.06) | 0.754 |
| POD6 | -12.90 (-19.50, -6.31) | -11.51 (-18.46, -4.57) | 0.776 | -9.53 (-14.23, -4.83) | -8.57 (-13.52, -3.62) | 0.783 |
| POD7 | -21.18 (-28.59, -13.77) | -11.75 (-19.76, -3.73) | 0.09 | -14.61 (-19.89, -9.33) | -8.24 (-13.96, -2.52) | 0.108 |
| 1-7 Days | -11.93 (-16.99, -6.88) | -11.97 (-17.03, -6.92) | 0.991 | -10.40 (-14.00, -6.79) | -9.62 (-13.23, -6.02) | 0.766 |
| 3 months | -13.75 (-21.44, -6.06) | -12.99 (-21.42, -4.56) | 0.895 | -11.02 (-16.50, -5.54) | -10.55 (-16.56, -4.54) | 0.909 |
| 6 months | -15.90 (-24.85, -6.94) | -15.22 (-22.20, -8.24) | 0.907 | -9.37 (-16.18, -2.55) | -6.18 (-11.15, -1.20) | 0.457 |
| 12 months | -10.73 (-17.33, -4.13) | -21.44 (-27.92, -14.97) | 0.022 | -6.51 (-11.11, -1.91) | -10.07 (-14.69, -5.45) | 0.282 |
| 5 years | Not estimable | Not estimable | - | Not estimable | Not estimable | - |
| 10 years | -15.72 (-28.74, -2.71) | -15.92 (-26.29, -5.55) | 0.982 | -12.63 (-21.91, -3.35) | -13.92 (-21.31, -6.53) | 0.831 |
|  |  |  | P for interaction = 0.311 |  |  | P for interaction = 0.490 |

Change over time in blood pressure post bariatric surgery by postoperative 1-year TWL% groups were estimated using maximum-likelihood linear mixed-effects models with participant-level random intercepts. Models were adjusted for age, sex, baseline BMI, baseline antihypertensive medication use, the corresponding baseline BP, baseline total cholesterol, study center, surgical procedures, and calendar year of surgery. The interaction term (time × postoperative 1-year TWL% group) was tested using Wald tests. BMI, body mass index; SBP, systolic blood pressure; DBP, diastolic blood pressure. LRYBG, laparoscopic Roux-en-Y geriatric bypass; LSG, laparoscopic sleeve gastrectomy; 95%CI = change from baseline 95% confidence interval.
Table S9 Inverse probability weighting-adjusted longitudinal changes in blood pressure after bariatric surgery.

| Time | Adjusted SBP change, mmHg (95% CI) | P value | Adjusted DBP change, mmHg (95% CI) | P value |
| --- | --- | --- | --- | --- |
| POD_1_ | -8.59 (-10.80, -6.39) | <0.001 | -11.02 (-12.79, -9.26) | <0.001 |
| POD_2_ | -6.88 (-8.94, -4.82) | <0.001 | -8.67 (-10.26, -7.08) | <0.001 |
| POD_3_ | -8.64 (-10.89, -6.38) | <0.001 | -9.68 (-11.34, -8.03) | <0.001 |
| POD_4_ | -10.25 (-12.53, -7.98) | <0.001 | -10.66 (-12.45, -8.88) | <0.001 |
| POD_5_ | -12.77 (-15.14, -10.40) | <0.001 | -12.40 (-14.37, -10.42) | <0.001 |
| POD_6_ | -12.56 (-15.23, -9.89) | <0.001 | -10.87 (-12.95, -8.79) | <0.001 |
| POD_7_ | -14.72 (-17.87, -11.57) | <0.001 | -12.56 (-15.16, -9.96) | <0.001 |
| 3 Months | -16.51 (-19.51, -13.50) | <0.001 | -11.19 (-13.39, -8.99) | <0.001 |
| 6 Months | -15.84 (-19.45, -12.23) | <0.001 | -8.92 (-11.92, -5.92) | <0.001 |
| 1 Years | -15.80 (-19.37, -12.23) | <0.001 | -8.87 (-11.55, -6.20) | <0.001 |
| 5 Years | -18.04 (-23.08, -13.01) | <0.001 | -9.56 (-12.53, -6.58) | <0.001 |
| 10 Years | -14.48 (-18.93, -10.04) | <0.001 | -11.11 (-15.73, -6.49) | <0.001 |

Changes in blood pressure over time following bariatric surgery, estimated using were an inverse probability weighting–adjusted mixed-effects models with participant-level random intercepts. Standard errors were estimated using robust variance clustered by participant. Models were adjusted for age, sex, baseline BMI, antihypertensive medication use, baseline blood pressure, baseline cholesterol, study center, surgical procedure, and calendar year of surgery.

Table S10. Multiple imputation sensitivity analysis for longitudinal changes in blood pressure after bariatric surgery.

| Time | Adjusted SBP change, mmHg (95% CI) | P value | Adjusted DBP change, mmHg (95% CI) | P value |
| --- | --- | --- | --- | --- |
| POD_1_ | -8.59 (-10.92, -6.25) | <0.001 | -11.02 (-12.81, -9.24) | <0.001 |
| POD_2_ | -6.54 (-9.01, -4.07) | <0.001 | -8.60 (-10.46, -6.74) | <0.001 |
| POD_3_ | -7.93 (-10.48, -5.38) | <0.001 | -9.27 (-11.16, -7.39) | <0.001 |
| POD_4_ | -9.46 (-11.91, -7.02) | <0.001 | -9.89 (-11.77, -8.00) | <0.001 |
| POD_5_ | -10.69 (-13.15, -8.23) | <0.001 | -10.22 (-12.11, -8.33) | <0.001 |
| POD_6_ | -11.18 (-14.56, -7.81) | <0.001 | -10.01 (-12.59, -7.43) | <0.001 |
| POD_7_ | -12.13 (-14.71, -9.54) | <0.001 | -10.38 (-12.44, -8.31) | <0.001 |
| 1-7 Days | -8.62 (-10.95, -6.29) | <0.001 | -10.43 (-12.21, -8.66) | <0.001 |
| 3 Months | -14.77 (-17.35, -12.18) | <0.001 | -11.30 (-13.20, -9.40) | <0.001 |
| 6 Months | -14.98 (-18.51, -11.46) | <0.001 | -11.76 (-14.01, -9.51) | <0.001 |
| 1 Years | -16.10 (-19.06, -13.13) | <0.001 | -12.88 (-15.81, -9.96) | <0.001 |
| 5 Years | -16.49 (-19.99, -12.98) | <0.001 | -11.45 (-13.63, -9.26) | <0.001 |
| 10 Years | -17.39 (-20.65, -14.14) | <0.001 | -13.34 (-15.69, -10.99) | <0.001 |

Changes in blood pressure over time following bariatric surgery, estimated using a linear mixed-effects model after multiple interpolations. Models were adjusted for age, sex, baseline BMI, baseline antihypertensive medication use, baseline blood pressure (SBP or DBP), baseline total cholesterol, hospital, and calendar year of surgery. Negative values indicate reductions in blood pressure relative to baseline.

Table S11 Long-term effect of bariatric surgery on other metabolic parameters.

| Variables | Preoperative | 3 Months | 6 Months | 1 Years | 5 Years | 10 Years |
| --- | --- | --- | --- | --- | --- | --- |
| BMI, kg/m^2^ | 38.04 ± 7.33 | 31.3 ± 5.7* | 25.77 ± 3.24* | 26 ± 5.79* | 30.05 ± 5.83* | 28.09 ± 5.27* |
| EWL, % | - | 70.99 ± 40.24* | 95.23 ± 45.93* | 106.51 ± 47. 01* | 71.05 ± 37.21* | 91.32 ± 55.95* |
| FBG, mmol/L | 7.62 ± 3.43 | 5.31 ± 1.66* | 5.06 ± 0.93* | 5.48 ± 1.13* | - | - |
| HbA1c, % | 7.39 ± 1.91 | 5.74 ± 0.96* | 5.73 ± 0.78* | 5.72 ± 0.78* | - | - |
| Fasting insulin, μU/ml | 28.71 ± 24.44 | 11.64 ± 11.27* | 7.62 ± 5.92* | 9.36 ± 6.41* | - | - |
| TC, mmol/L | 4.90 ± 1.04 | 4.32 ± 1.11* | 4.17 ± 1.04* | 4.07 ± 0.96* | - | - |
| TG, mmol/L | 2.64 ± 2.24 | 1.35 ± 0.58* | 1.30 ± 0.52* | 1.08 ± 0.45* | - | - |
| HDL, mmol/L | 1.06 ± 0.26 | 1.11 ± 0.4* | 1.12 ± 0.32* | 1.34 ± 0.48* | - | - |
| LDL, mmol/L | 2.84 ± 0.88 | 2.64 ± 0.89* | 2.60 ± 0.91* | 2.42 ± 0.67* | - | - |

BMI, body mass index; EWL, excess weight loss; TC, total cholesterol; TG, triglyceride; FBG, fasting blood glucose.

^*^P<0.001, compared with preoperative parameters.

Table S12 Preoperative characteristics of patients in mechanism analysis.

| Variables | Patients in the mechanism analysis (n=14) | Hypertensive (n=248) | P value |
| --- | --- | --- | --- |
| Age, y | 32.5 ± 10.38 | 33.67 ± 11.13 | 0.147 |
| Gender, male, n (%) | 9 (64.29%) | 123 (49.60%) | 0.411 |
| BMI, kg/m^2^ | 39.06 ± 4.02 | 38.04 ± 7.33 | 0.609 |
| WC, cm | 114.36 ± 10.67 | 118.15 ± 15.97 | 0.381 |
| SBP, mmHg | 149.43 ± 9.06 | 142.7 ± 13.96 | 0.076 |
| DBP, mmHg | 98.36 ± 11.76 | 93.93 ± 10.94 | 0.143 |

BMI, body mass index; SBP, systolic blood pressure; DBP, diastolic blood pressure. WC, waist circumference.

Table S13 Baseline mechanistic biomarker levels and paired individual-level changes from baseline following bariatric surgery.

| Variables | Preoperative (n=14) | Day 3 mean difference (95% CI, n=12) | P value^*^ | FDR-adjusted q value | Day 6 mean difference (95% CI, n=8) | P value^*^ | FDR-adjusted q value |
| --- | --- | --- | --- | --- | --- | --- | --- |
| Leptin, ng/ml | 6.93 (5.32, 9.39) | -0.71 (-3.07, 1.66) | 0.814 | 0.885 | -3.52 (-6.39, -0.66) | 0.123 | 0.294 |
| Adiponectin, ng/ml | 10880.72 ± 5422.345 | 544.46 (-1836.71, 2925.63) | 0.625 | 0.775 | 1988.18 (-929.31, 4905.67) | 0.151 | 0.312 |
| Ghrelin, pg/ml | 2845.25 ± 1982.36 | 158.61 (-479.54, 796.76) | 0.595 | 0.759 | -22.64 (-943.37, 898.10) | 0.955 | 0.955 |
| Peptide YY, pg/ml | 76.94 (73.35, 93.01) | -6.75 (-18.19, 4.69) | 0.433 | 0.661 | -3.40 (-37.50, 30.71) | 0.674 | 0.789 |
| GLP-1, ng/ml | 6.24 (3.92, 12.17) | -1.42 (-5.63, 2.78) | 0.023^*^ | 0.201 | -3.82 (-7.53, -0.11) | 0.012^*^ | 0.182 |
| ANP, pg/ml | 49.81 (19.44, 129.86) | 20.68 (-202.88, 244.23) | 0.53 | 0.747 | -63.34 (-214.33, 87.65) | 0.093 | 0.25 |
| ADH, pg/ml | 9.70 ± 4.79 | -0.91 (-4.17, 2.35) | 0.552 | 0.759 | 1.44 (-5.99, 8.86) | 0.661 | 0.788 |
| Renin, standing, ng/ml/h | 4.44 (3.17, 11.84) | -1.09 (-4.88, 2.71) | 0.695 | 0.798 | -0.72 (-7.97, 6.52) | 0.917 | 0.932 |
| Renin, lying, ng/ml/h | 1.45 (1.06, 3.42) | -0.39 (-1.32, 0.55) | 0.062 | 0.234 | -0.44 (-2.72, 1.85) | 0.463 | 0.668 |
| Ang-1, standing, ng/ml/h | 3.02 (2.14, 5.38) | -0.60 (-1.76, 0.56) | 0.136 | 0.301 | 0.63 (-1.73, 2.99) | 0.463 | 0.668 |
| Ang-1, lying, ng/ml/h | 3.71 (1.85, 4.20) | -0.51 (-1.96, 0.95) | 0.068 | 0.234 | -0.92 (-3.09, 1.26) | 0.249 | 0.441 |
| Ang-2, standing, pg/ml/h | 54.20 (33.68, 38.87) | -13.25 (-29.56, 3.06) | 0.136 | 0.301 | 5.74 (-18.72, 30.20) | 0.046^*^ | 0.201 |
| Ang-2, lying, pg/ml/h | 44.89 (32.15, 69.85) | -4.78 (-18.56, 9.00) | 0.075 | 0.234 | 9.13 (-14.01, 32.26) | 0.345 | 0.549 |
| Aldosterone, standing, pg/ml/h | 253.55 ± 105.70 | -59.75 (-123.70, 4.20) | 0.099 | 0.257 | 34.50 (-95.10, 164.10) | 0.6 | 0.759 |
| Aldosterone, lying, pg/ml/h | 194.75 ± 84.96 | -31.30 (-119.99, 57.39) | 0.328 | 0.535 | 40.61 (-48.12, 129.33) | 0.917 | 0.932 |
| Dopamine, ng/ml | 3.32 (1.93, 8.59) | 0.08 (-2.07, 2.23) | 0.754 | 0.85 | 0.10 (-5.34, 5.55) | 0.889 | 0.932 |
| Epinephrine, pg/ml | 91.90 (39.78, 193.87) | -7.56 (-22.89, 7.77) | 0.239 | 0.436 | 23.12 (-128.67, 174.90) | 0.779 | 0.863 |
| Norepinephrine, pg/ml | 3.93 (2.67, 14.57) | 0.34 (-4.35, 5.04) | 0.583 | 0.759 | 0.46 (-10.02, 10.93) | 0.327 | 0.535 |
| 24-h urine volume, ml | 1250.00 ± 536.03 | 1398.75 (525.66, 2271.84) | 0.007^*^ | 0.141 | 1634.29 (782.39, 2486.18) | 0.003^*^ | 0.104 |
| 24-h urine Na^+^, mmol/day | 229.60 ± 176.49 | 512.18 (148.75, 875.61) | 0.013^*^ | 0.111 | 595.12 (182.63, 1007.60) | 0.012^*^ | 0.111 |
| 24-h urine Cl^-^, mmol/day | 196.11 ± 148.03 | 462.42 (137.07, 787.76) | 0.012^*^ | 0.111 | 655.08 (122.10, 1188.06) | 0.024^*^ | 0.164 |
| 24-h urine K^+^, mmol/day | 61.37 ± 26.70 | 70.87 (2.29, 139.44) | 0.045^*^ | 0.168 | 205.74 (-88.90, 500.37) | 0.138 | 0.286 |
| Total water content, kg/m^2^ | 47.32 ± 7.69 | -1.54 (-3.23, 0.15) | 0.068 | 0.234 | -6.92 (-17.79, 3.95) | 0.17 | 0.34 |
| Intracellular water content, L | 29.52 ± 5.00 | -0.84 (-1.64, -0.05) | 0.039^*^ | 0.201 | -1.40 (-2.74, -0.06) | 0.044^*^ | 0.201 |
| Exocellular water content, L | 17.81 ± 2.79 | -0.71 (-1.63, 0.20) | 0.111 | 0.275 | -1.07 (-2.29, 0.15) | 0.075 | 0.234 |

Continuous variables with a normal distribution were reported as mean ± SD (standard deviation); continuous variables with a non-normal distribution were reported as medians and IQR (interquartile ranges). Early changes values are presented as mean change (95% CI) from baseline for normally distributed biomarkers and median change (95% CI) for skewed biomarkers. P values were derived from paired t tests or Wilcoxon signed-rank tests as appropriate. Multiple comparisons were controlled using the Benjamini-Hochberg false discovery rate (FDR) method.

GLP-1, glucagon-like peptide-1; ANP, A-type natriuretic peptide; ADH, antidiuretic hormone; Ang-1, angiotensin-1; Ang-2, angiotensin-2.

^*^ Compared with preoperative indices.

Table S14 Baseline characteristics in total, hypertensive, and non-hypertensive patients.

| Variables | Total (n=454) | Hypertensive (n=248) | Non-hypertensive (n=206) | P value |
| --- | --- | --- | --- | --- |
| Age, y | 36.83 ± 10.86 | 36.84 ± 10.86 | 35.25 ± 2.24 | 0.002 |
| Gender, Female, n (%) | 257 (56.61%) | 123 (49.60%) | 134 (65%) | 0.001 |
| BMI, kg/m^2^ | 37.17 ± 1.23 | 38.04 ± 7.33 | 36.3 ± 7.56 | 0.013 |
| WC, cm | 115.86 ± 3.24 | 118.15 ± 15.97 | 113.57 ± 17.34 | 0.004 |
| SBP, mmHg | 132.62 ± 14.25 | 142.7 ± 13.96 | 122.55 ± 9.38 | <0.001 |
| DBP, mmHg | 85.86 ± 11.4 | 93.93 ± 10.94 | 77.8 ± 7.09 | <0.001 |
| Hb, g/L | 142.58 ± 3.56 | 145.1 ± 17 | 140.06 ± 17.24 | 0.002 |
| RBC count, 10^12^/L | 4.92 ± 0.13 | 5.02 ± 0.76 | 4.83 ± 0.61 | 0.004 |
| Albumin, g/L | 44.29 ± 0.26 | 44.48 ± 4.14 | 44.11 ± 4.61 | 0.381 |
| Scr, mmol/L | 64.55 ± 2.48 | 66.31 ± 20.1 | 62.8 ± 15.54 | 0.042 |
| UA, mmol/L | 402.51 ± 11.67 | 410.76 ± 111.11 | 394.25 ± 107.6 | 0.116 |
| TC, mmol/L | 4.89 ± 0.03 | 4.9 ± 1.04 | 4.87 ± 1.27 | 0.738 |
| TG, mmol/L | 2.52 ± 0.17 | 2.64 ± 2.24 | 2.4 ± 3.29 | 0.347 |
| HDL-C, mmol/L | 1.06 ± 0.01 | 1.06 ± 0.26 | 1.05 ± 0.3 | 0.665 |
| LDL-C, mmol/L | 2.9 ± 0.08 | 2.84 ± 0.88 | 2.96 ± 1.05 | 0.204 |
| FBG, mmol/L | 7.27 ± 0.49 | 7.62 ± 3.43 | 6.92 ± 3.16 | 0.028 |
| Fasting insulin, μU/ml | 28.07 ± 0.91 | 28.71 ± 24.44 | 27.43 ± 20.63 | 0.586 |
| HbA1c, % | 7.14 ± 0.35 | 7.39 ± 1.91 | 6.89 ± 1.99 | 0.012 |
| Use of antihypertensive medications, n (%) | 84 (18.5%) | 84 (33.8%) | 0 (0%) | <0.001 |

BMI, body mass index; WC, waist circumference; SBP, systolic blood pressure; DBP, diastolic blood pressure; Hb, hemoglobin; RBC, red blood cell; Scr, serum creatinine; UA, uric acid; TC, total cholesterol; TG, triglyceride; HDL-C, high-density lipoprotein cholesterol; LDL-C, low-density lipoprotein cholesterol; FBG, fasting blood glucose; BP, blood pressure.

Continuous variables with a normal distribution were reported as mean ± SD (standard deviation), and the group comparisons were tested by t-test. Continuous variables with a non-normal distribution were reported as medians and IQR (interquartile ranges), and the group comparisons were tested by the Kruskal-Wallis test. Categorical variables were tested by the Chi-square test.

Table S15 Guideline-based indications and contraindications for bariatric/metabolic surgery in China (2007–2019).

| Guideline (Year) | Applicable period in the study | Indications for surgery | Contraindications to surgery/anesthesia |
| --- | --- | --- | --- |
| Chinese Guidelines for Surgical Treatment of Obesity (2007) ^1^ | 2010–2013 | • Presence of metabolic disorders related to excessive adiposity, such as type 2 diabetes, cardiovascular disease, fatty liver, dyslipidemia, or obstructive sleep apnea, is expected to improve after weight reduction. • Waist circumference: ≥90 cm (men), ≥80 cm (women) with lipid abnormalities (TG ≥1.7 mmol/L and/or HDL-C <0.9 mmol/L in men or <1.0 mmol/L in women). • BMI ≥32 kg/m², or progressive weight gain for ≥5 years (excluding transient pregnancy-related weight changes). • Age 16–65 years; patients <16 or >65 years require individualized assessment of risks and benefits. • Ineffective or intolerant to non-surgical therapy. • No alcohol or drug dependence, no severe psychiatric or intellectual disorder. • Fully understands surgical procedures and potential complications, and is willing and able to comply with postoperative lifestyle changes and long-term follow-up. | Patients not meeting the above conditions are not recommended for bariatric surgery. |
| Chinese Guidelines for Bariatric and Metabolic Surgery (2014) ^2^ | 2014–2018 | • T2DM duration ≤ 15 years with residual β-cell function (fasting C-peptide ≥ 50% of lower limit of normal). • BMI-based criteria: see Table 1 of guideline; generally, BMI ≥ 32.5 kg/m², or BMI ≥ 27.5 kg/m² with poorly controlled T2DM or obesity-related comorbidities despite optimal medical therapy. • Waist circumference: ≥90 cm (men), ≥85 cm (women) increases the surgical recommendation level. • Age 16–65 years. • Multidisciplinary team (MDT) evaluation confirming eligibility. | • Non-obese type 1 diabetes or β-cell failure (C-peptide absent). • BMI < 25 kg/m² (not recommended). • Gestational diabetes or special diabetes types. • Active substance abuse or alcohol dependence. • Uncontrolled psychiatric illness or cognitive immaturity. • Unrealistic expectations or refusal to accept surgical risks. • Poor compliance with postoperative dietary and lifestyle requirements. • Severe systemic disease intolerant to anesthesia or surgery. |
| Chinese Guidelines for Bariatric and Metabolic Surgery (2019 Edition) ^3^ | 2019 onward | For obesity: • BMI ≥ 37.5 kg/m² – surgery strongly recommended; BMI 32.5–37.5 – recommended; BMI 27.5–32.5 – consider after failed lifestyle/medical therapy and ≥2 metabolic syndrome components or comorbidities. • Central obesity (waist ≥ 90 cm men, ≥ 85 cm women) may increase recommendation grade after MDT discussion. • Age 16–65 years (individualized if outside range).  For T2DM: • Residual β-cell function present. • BMI ≥ 32.5 kg/m² – strongly recommended; BMI 27.5–32.5 – recommended; BMI 25–27.5 – consider cautiously if ≥2 metabolic syndrome components or comorbidities and poor glycemic control. • Central obesity (waist ≥ 90 cm men, ≥ 85 cm women) can upgrade surgical recommendation after MDT review. | • Non-obese type 1 diabetes. • T2DM patients with absent β-cell function. • BMI < 25 kg/m² – not recommended. • Gestational or specific types of diabetes. • Active substance or alcohol dependence. • Uncontrolled psychiatric illness or cognitive immaturity. • Unrealistic expectations or refusal to accept surgical risk. • Poor compliance with postoperative care. • Poor general condition, intolerant to anesthesia or surgery. |

**Reference**

1.	Chinese Society of Surgery - Endocrine Surgery Group, Laparoscopic and Endoscopic Surgery Group‌, Gastroenterological Surgery Group, Chinese Society of Surgery - **Surgical Operation Group.** Chinese Guidelines for Surgical Treatment of Obesity (2007 Edition). *Chinese Journal of Practical Surgery*. 2007;27(10):759-762.

2.	Chinese Society for Metabolic & Bariatric Surgery. Chinese Guidelines for Bariatric and Metabolic Surgery (2014 Edition). *Chinese Journal of Practical Surgery*. 2014;34(11):1005-1010.

3.	Thyroid and Metabolic Surgery Group of the Chinese Society of Surgery‌, Chinese Society for Metabolic & Bariatric Surgery. Chinese Guidelines for Bariatric and Metabolic Surgery (2019 Edition). *Chinese Journal of Practical Surgery*. 2019;39(4):301-306.
